# Super-enhancers: regulatory mechanisms, biological functions, diseases, and therapeutic targets

**DOI:** 10.1186/s43556-026-00647-6

**Published:** 2026-11-02

**Authors:** Huijie Miao, Yuanding Liu, Kaini Yang, Ao Gu, Cuifeng Zhang, Mengyao Li, Jie Wang

**Affiliations:** 1 Department of Breast Surgery, The International Peace Maternity and Child Health Hospital, Shanghai Jiao Tong University School of Medicine, Shanghai, China; 2 State Key Laboratory of Systems Medicine for Cancer, Shanghai Cancer Institute, Renji Hospital, Shanghai Jiao Tong University School of Medicine, Shanghai, China; 3 Department of Biliary-Pancreatic Surgery, Ren Ji Hospital, Shanghai Jiao Tong University School of Medicine, Shanghai, China; 4 Shanghai Key Laboratory for Cancer Systems Regulation and Clinical Translation, Shanghai Jiading District Central Hospital, Shanghai, China

**Keywords:** Super-enhancers, Transcriptional regulation, Chromatin organization, Human diseases, Breast cancer, Therapeutic targeting

## Abstract

Super-enhancers (SEs) are operationally defined as clusters of cis-regulatory elements associated with high transcriptional activity and cell-identity genes. They display dense transcription factor and coactivator occupancy, strong H3K27 acetylation, and enhancer–promoter communication, but their annotation varies across platforms, algorithms, and thresholds. This review examines SE architecture, identification, and validation, distinguishing chromatin-based annotation from functional dependence demonstrated by locus-specific perturbations. Mechanisms of SE-associated transcription include pioneer factors, chromatin remodelers, three-dimensional genome organization, transcriptional condensates, non-coding RNAs, and extrachromosomal DNA. Physiological functions in development, immunity, and metabolic homeostasis are considered alongside context-dependent dysregulation in cancer, inflammatory and autoimmune diseases, neurological disorders, and metabolic diseases. Breast cancer provides a model for examining subtype-specific regulation and transcriptional plasticity. Particular attention is given to enhancer remodeling during endocrine therapy, cyclin-dependent kinase 4/6 (CDK4/6) inhibition, human epidermal growth factor receptor 2 (HER2)-targeted treatment, and chemotherapy, which may support bypass signaling, stemness, and persistent resistance. Causal regulatory changes must nevertheless be distinguished from correlates of treatment selection. Therapeutic approaches targeting bromodomain and extraterminal domain (BET) proteins, CDK7/9, chromatin regulators, enhancer RNAs, and regulatory loci are critically evaluated. Definitional uncertainty, model dependence, limited reproducibility, systemic toxicity, and insufficient clinical validation remain major barriers. Priorities include standardized annotation, temporally resolved perturbation, single-cell and spatial profiling, artificial intelligence-assisted integration, patient-derived models, and biomarker-guided therapeutic development. Together, these perspectives position SEs as a context-dependent framework linking regulatory architecture to disease progression and treatment resistance, while emphasizing the evidence required to establish therapeutic vulnerabilities.

## Introduction

Precise control of gene expression relies on the coordinated actions of transcription factors, chromatin regulators and non-coding cis-regulatory elements that shape chromatin accessibility and three-dimensional genome organization [1–3]. The term super-enhancer was introduced in 2013 by studies identifying unusually large enhancer domains with dense occupancy of lineage-defining transcription factors and Mediator at genes central to cell identity [4, 5]. Super-enhancers (SEs) are generally described as clusters of constituent enhancers characterized by extensive cofactor recruitment and high levels of active chromatin modifications, particularly histone H3 lysine 27 acetylation (H3K27ac) [4, 5]. This concept has provided a useful framework for identifying regulatory domains with strong transcriptional activity and cell-type specificity. However, SEs are commonly classified using relative epigenomic signals and ranking algorithms rather than universal fixed biological criteria [6–8]. Because enhancer activity often varies along a quantitative continuum, SE calls are sensitive to experimental platform, peak-calling strategy, stitching distance, signal normalization and threshold selection [6–8]. Therefore, SEs should be interpreted as context-dependent regulatory domains whose biological relevance requires functional evaluation, rather than as a uniform discrete class of enhancers.

Beyond their association with highly expressed genes, SEs have been implicated in lineage specification, embryonic development, immune regulation and metabolic homeostasis [5, 9–11]. Their activity is shaped by lineage-defining and signal-responsive transcription factors, enhancer–promoter communication, coactivator condensation and non-coding RNA-dependent regulation, allowing extracellular signals, chromatin states and nuclear organization to be integrated into cell-state-specific transcriptional outputs [4, 12–14]. Reconfiguration of these regulatory systems has been associated with cancer and other diseases where cellular identity or adaptive transcription is disrupted [5, 15]. Breast cancer provides an informative model where estrogen receptor alpha (ERα)-associated and triple-negative breast cancer-specific SE programs have been identified and functionally investigated [16, 17]. In contrast, ERBB2-associated enhancers have been reported, but the classification as a functionally validated ERBB2-associated SE and its remodeling during human epidermal growth factor receptor 2 (HER2)-targeted therapy remain less well established [18]. These observations illustrate an important distinction: disease-associated SE signatures may identify altered transcriptional states but do not necessarily establish the regulatory dependencies required to maintain the corresponding phenotype.

Despite substantial progress, several conceptual and translational challenges continue to limit the interpretation of SE biology. Many SE annotations and SE–gene assignments remain correlative and require validation through locus-specific genetic or epigenetic perturbation, physiologically relevant models and clinically annotated datasets [6–8, 19, 20]. Moreover, pharmacological approaches directed at SE-associated transcription generally engage broadly used chromatin and transcriptional regulators, raising concerns regarding selectivity, systemic toxicity and therapeutic windows [15, 21]. Emerging approaches—including single-cell and spatial multi-omics, chromatin conformation analysis, clustered regularly interspaced short palindromic repeats (CRISPR)-based perturbation and computational integration—provide opportunities to refine SE annotation and identify context-specific regulatory dependencies [8, 12, 19, 20, 22–25]. An integrated assessment is therefore needed to distinguish descriptive SE maps from functionally validated regulatory dependencies, reconcile evidence across physiological and disease contexts, and critically evaluate the translational potential of SE-associated vulnerabilities. In this review, we first examine the structural, functional and methodological frameworks for SE identification, target-gene assignment and functional validation across complementary experimental models. We then discuss the molecular mechanisms and physiological functions of SEs, their dysregulation across human diseases—with in-depth consideration of breast cancer—and the evidence and limitations surrounding therapeutic intervention. Finally, we integrate therapeutic targeting with treatment-induced SE reprogramming and resistance, critically appraising current translational barriers and prioritizing actionable strategies to render SE research more reproducible, mechanistically grounded, and clinically informative.

## Structural, functional, and methodological framework of SEs

### Structural organization and molecular features of SEs

**Fig. 1:**
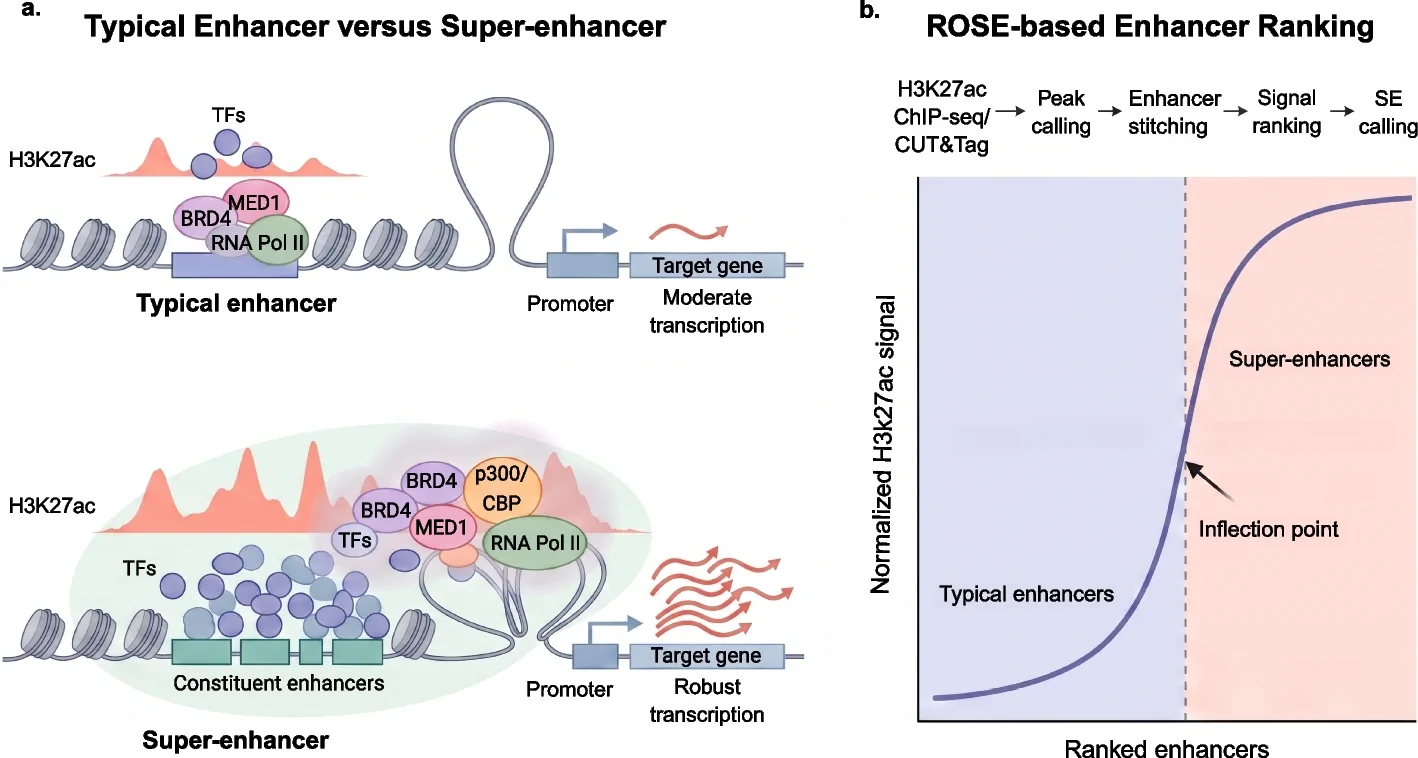
Structural features and operational identification of SEs. **a** Typical enhancers recruit transcription factors (TFs), coactivators and RNA polymerase II to promote moderate target-gene transcription through enhancer–promoter looping. SEs comprise clusters of constituent enhancers characterized by strong H3K27ac enrichment and dense occupancy by TFs, BRD4, MED1, p300, CBP and RNA polymerase II. The schematic illustrates their association with robust transcription. **b** In the Rank Ordering of Super-Enhancers (ROSE) workflow, H3K27ac-enriched regions identified by ChIP–seq or CUT&Tag undergo peak calling, stitching of neighboring enhancers, and ranking by normalized signal. A dataset-relative cutoff on the ranking curve operationally separates SE candidates from typical enhancers. The dashed line marks the operational threshold used for SE calling. This classification depends on analytical parameters and does not, by itself, establish functional dependence or identify target genes. *Created in BioRender. Miao, H. (2026) *https://BioRender.com/zw0m8xm

Super-enhancers (SEs) are operationally defined regulatory domains comprising clusters of enhancer elements with exceptionally high aggregate occupancy by transcription factors, coactivators, and strong enrichment of active chromatin marks, most commonly H3K27ac [4, 5, 7, 8, 26] (Fig. 1). They are not specified by a unique DNA sequence, fixed size or invariant molecular composition. In commonly used annotation workflows, nearby candidate enhancers are stitched and ranked according to their aggregate chromatin signal. The resulting domains generally exhibit broader and stronger regulatory signals than typical enhancers, although their inferred boundaries depend on the experimental assay, biological context and analytical parameters. SEs should therefore be regarded as a context-dependent operational category rather than a fixed anatomical feature of the genome. Their constituent composition can vary across cell types and cellular states, which limits direct comparisons between SE maps generated in different biological systems.

At the molecular level, SEs typically encompass accessible chromatin, dense clusters of transcription-factor binding sites, active histone modifications, RNA polymerase occupancy, enhancer transcription and long-range contacts with target promoters [27]. Mediator complex subunit 1 (MED1), bromodomain-containing protein 4 (BRD4) and other cofactors can accumulate at these domains and engage in multivalent interactions that concentrate components of the transcriptional machinery [13, 28, 29]. Such condensate-like organization may facilitate transcription at selected loci, but it is neither universal nor sufficient to define an SE. Constituent elements are also functionally heterogeneous. Genetic dissection has identified classical enhancers with intrinsic activity, partially redundant or hierarchically organized elements, and facilitators that augment neighboring enhancers without acting as strong autonomous activators [6, 30, 31]. Accordingly, domain size or chromatin-signal intensity alone cannot establish which constituent elements are functionally indispensable. An SE is therefore better viewed as a context-dependent regulatory ensemble than as a uniform collection of equivalent enhancers. Its transcriptional output reflects the combined effects of local element composition, cofactor availability, three-dimensional genome organization and promoter responsiveness. This architecture may confer transcriptional robustness in some contexts while creating dependence on a limited subset of regulatory elements in others.

### Functional properties and biological roles of SEs

SEs are frequently associated with genes that establish cell identity and support high-output or rapidly inducible transcriptional programs [4, 5]. This association, however, should not be equated with functional dependence. Their regulatory importance extends beyond transcriptional magnitude and may reflect their capacity to integrate lineage-defining transcription factors with developmental, hormonal, inflammatory and metabolic signals [9–11]. At some loci, clustered enhancers provide transcriptional robustness through partially redundant inputs; at others, cooperative or hierarchical interactions confer heightened responsiveness to changes in signaling or transcription-factor availability [6, 30, 31]. These behaviors are context dependent and cannot be inferred from H3K27ac intensity or domain size alone. Regulatory output is further shaped by promoter compatibility, enhancer–promoter contact frequency and the insulating properties of the surrounding chromatin domain [12, 27, 32]. Consequently, similarly annotated SEs may exert distinct quantitative or gene-specific effects across cell types and physiological states.

Functional testing is therefore essential for distinguishing chromatin-defined SEs from regulatory dependencies. Constituent-element deletion can produce effects ranging from minimal changes to near-complete loss of target-gene expression, depending on the degree of redundancy and hierarchical organization within the domain [6, 30, 31]. Endogenous deletion, CRISPR interference and locus-specific epigenome editing can test the necessity of a candidate SE or individual constituent, whereas activation-based approaches can help assess sufficiency [20, 32]. These perturbations should be combined with transcriptional measurements and chromatin-contact analysis to distinguish direct target genes from genes associated only by genomic proximity. In normal tissues, functionally supported SE-associated circuits contribute to development, immune-cell specification and metabolic homeostasis [5, 9–11]. In disease, oncogenic transcription factors, altered chromatin regulators and aberrant signaling can redirect the same regulatory logic toward programs that support proliferation, lineage plasticity and survival [15, 33]. SE maps can therefore identify cell-state markers and candidate vulnerabilities, but annotation alone does not establish a therapeutic target. Translational relevance requires evidence that the dependency is causal, maintained in clinically relevant models and sufficiently selective to be separated from broadly required transcriptional processes.

### Methodological framework for SE identification and functional validation

SE analysis involves three distinct tasks: nominating candidate domains, assigning their target genes, and testing regulatory function. Chromatin activity and cofactor occupancy support the initial annotation; transcriptional and three-dimensional genome data then narrow the possible target genes. Genetic or epigenetic perturbation is required to determine whether the candidate domain is necessary or sufficient. Cell lines, organoids, animal models, and patient-derived material address different aspects of biological and translational relevance. The subsections below follow this sequence and then consider the limitations that affect reproducibility.

#### Integrated approaches for SE identification, target-gene assignment, and functional validation

**Fig. 2:**
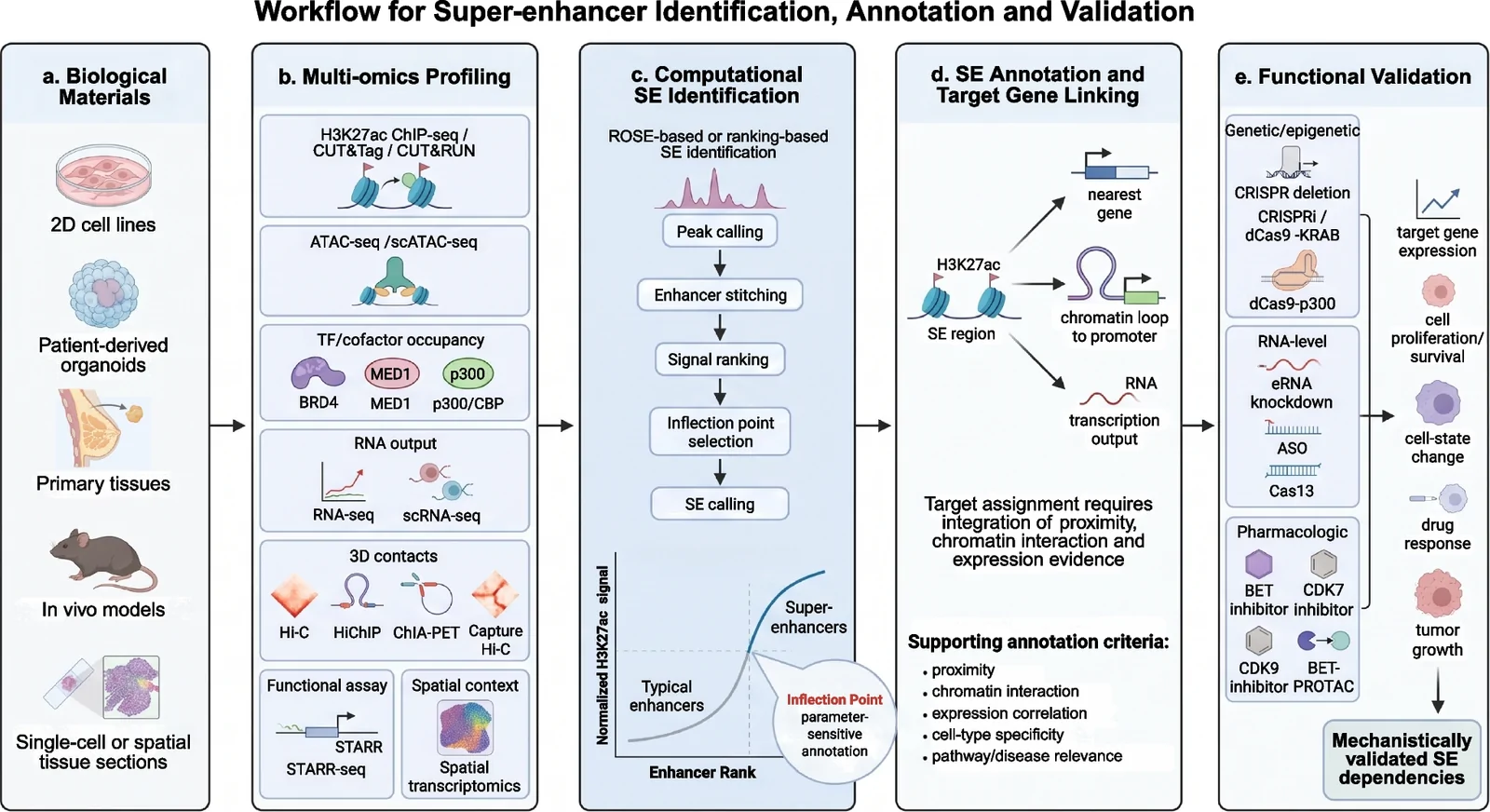
Workflow for super-enhancer identification, annotation and functional validation. **a** Biological materials used to investigate super-enhancers (SEs), including two-dimensional cell lines, patient-derived organoids, primary tissues, in vivo models, and specimens for single-cell or spatial analyses. These complementary systems provide different levels of biological complexity and support the investigation of SE regulation in defined cellular and tissue contexts. **b** Multi-omics profiling and complementary assays for characterizing candidate SEs. H3K27ac profiling by ChIP-seq, CUT&Tag, or CUT&RUN identifies active regulatory regions, while ATAC-seq and single-cell ATAC-seq assess chromatin accessibility. Profiling the occupancy of transcription factors and cofactors, such as BRD4, MED1, and p300/CBP, provides additional regulatory information. Bulk and single-cell RNA sequencing measure transcriptional output, whereas Hi-C, HiChIP, ChIA-PET, and Capture Hi-C characterize three-dimensional chromatin contacts. STARR-seq assesses reporter-based enhancer activity, and spatial transcriptomics places gene expression within the tissue context. **c** Computational identification of SEs using Rank Ordering of Super-Enhancers (ROSE) or related ranking approaches. The workflow comprises peak calling, stitching of neighboring enhancer regions, ranking by regulatory signal, selection of an inflection-point threshold, and SE calling. The schematic ranking curve illustrates the separation of typical enhancers from high-signal SE candidates. This classification is sensitive to analytical parameters and represents an operational annotation rather than direct evidence of functional dependence. **d** SE annotation and assignment of candidate target genes through the integration of genomic proximity, enhancer–promoter chromatin interactions, and expression evidence. The nearest gene is considered a candidate rather than an automatically established target. Additional supporting information includes expression correlations, cell-type specificity, and pathway or disease relevance. Integrating these features guides the prioritization of SE–gene relationships for experimental testing. **e** Functional assessment of candidate SE dependencies through genetic, epigenetic, RNA-directed, and pharmacological perturbations. Approaches include CRISPR-mediated deletion, CRISPR interference using dCas9–KRAB, targeted epigenetic activation using dCas9–p300, and enhancer RNA depletion using antisense oligonucleotides or Cas13. Pharmacological strategies include BET, CDK7, and CDK9 inhibitors and BET-directed proteolysis-targeting chimeras. Readouts include target-gene expression, cell proliferation and survival, cell-state changes, drug responses, and tumor growth. Pharmacological responses are interpreted alongside locus-specific and RNA-directed perturbations to distinguish broader transcriptional effects from mechanistically supported SE dependencies. *Created in BioRender. Miao, H. (2026) *https://BioRender.com/zw0m8xm

The identification and characterization of SEs have been enabled by advances in high-throughput genomic and epigenomic technologies that capture chromatin states, regulatory activity and three-dimensional genome organization (Fig. 2). Chromatin immunoprecipitation followed by sequencing (ChIP–seq) remains the most widely used approach for SE annotation, with histone H3 lysine 27 acetylation (H3K27ac) serving as a key marker of active enhancer regions [34] (Table 1). Computational frameworks, most notably the Rank Ordering of Super-Enhancers (ROSE) algorithm, integrate ChIP–seq signal intensity and genomic proximity to distinguish SEs from typical enhancers. Complementary assays, including assay for transposase-accessible chromatin using sequencing (ATAC–seq), provide information on chromatin accessibility and facilitate the identification of regulatory regions permissive for transcription factor binding [38]. More recently, low-input chromatin profiling methods such as cleavage under targets and release using nuclease (CUT&RUN) and cleavage under targets and tagmentation (CUT&Tag) have improved the resolution and sensitivity of chromatin mapping, enabling the profiling of histone modifications and transcription factor occupancy from limited cellular material [36, 37]. Functional validation of candidate SEs has been further facilitated by self-transcribing active regulatory region sequencing (STARR–seq), which enables quantitative assessment of enhancer activity on a genome-wide scale and complements epigenomic approaches by directly evaluating regulatory function [39].

**Table 1:** Technologies for SE identification, annotation and functional validation

Technology	Primary readout	Principal application in SE research	Key strengths	Principal limitations	Functional evidence provided
H3K27ac ChIP-seq	Genome-wide H3K27ac occupancy	Operational SE identification and signal ranking	Established and widely benchmarked; compatible with ROSE	Conventional protocols require substantial input; antibody-, peak-calling- and threshold-dependent	Indirect [26, 34, 35]
CUT&Tag/CUT&RUN	Histone modification or regulatory-protein occupancy	Low-input SE profiling and cofactor mapping	Low input requirements; often high signal-to-background ratio	Antibody- and protocol-dependent; assay-specific biases may limit cross-study comparability	Indirect [36, 37]
ATAC-seq	Chromatin accessibility	Mapping candidate regulatory elements and accessible SE regions	Low input requirements; compatible with single-cell profiling	Accessibility does not establish enhancer activity or SE status	Indirect [38, 39]
STARR-seq	Enhancer-driven reporter transcription	High-throughput functional screening of candidate enhancer sequences	Quantitative assessment of autonomous enhancer activity	Episomal context lacks native chromatin and three-dimensional genome organization	Direct for sequence-intrinsic activity, but not endogenous SE dependency [39]
Hi-C	Genome-wide chromatin contacts	Defining regulatory domains and candidate SE-promoter interactions	Captures global three-dimensional genome architecture	Requires high sequencing depth; limited resolution; physical contact does not establish regulatory function	Supportive [32, 40]
HiChIP/ChIA-PET	Protein-associated chromatin contacts	Mapping SE-promoter interactions and supporting target-gene assignment	Enriches factor-associated regulatory contacts at relatively high resolution	Factor- and antibody-dependent; detected contacts are not necessarily functional	Supportive [41, 42]
scATAC-seq/scRNA-seq integration	Cell-resolved chromatin accessibility and transcription	Inferring cell-type-specific SE landscapes and transcriptional programs	Resolves cellular heterogeneity and state-specific regulation	Sparse data, batch effects and indirect SE-to-gene assignment	Indirect [23, 43, 44]
Spatial transcriptomics	Spatially resolved gene expression	Placing SE-associated transcriptional programs within tissue context	Preserves spatial organization and cellular neighborhoods	Does not directly measure enhancer activity; resolution and sensitivity vary by platform	Indirect [45, 46]
CRISPR deletion, CRISPRi/a or epigenome editing	Locus-specific perturbation and downstream molecular or phenotypic effects	Testing endogenous SE activity and regulatory dependency	Enables causal interrogation in native chromatin context	Off-target effects, incomplete perturbation and disruption of neighboring regulatory architecture	Direct when locus-specific effects are linked to target-gene and phenotypic changes [19, 20, 47]

*Abbreviations*: *ATAC-seq* assay for transposase-accessible chromatin using sequencing, *ChIA-PET* chromatin interaction analysis with paired-end tag sequencing, *ChIP-seq* chromatin immunoprecipitation followed by sequencing, *CRISPRa* CRISPR activation; CRISPRi, CRISPR interference, *CUT&RUN* cleavage under targets and release using nuclease, *CUT&Tag* cleavage under targets and tagmentation, *H3K27ac* histone H3 lysine 27 acetylation, *ROSE* rank ordering of super-enhancers, *SE* super-enhancer, *STARR-seq* self-transcribing active regulatory region sequencing, *Hi-C* high-throughput chromosome conformation capture, *HiChIP* Hi-C coupled with chromatin immunoprecipitation, *scATAC-seq* single-cell assay for transposase-accessible chromatin using sequencing, *scRNA-seq* single-cell RNA sequencing

The regulatory architecture of SEs is closely linked to higher-order chromatin organization. Chromosome conformation capture–based technologies have provided important insights into the spatial interactions that connect SEs with their target genes [41]. High-throughput chromosome conformation capture (Hi-C) has enabled genome-wide mapping of chromatin interactions, whereas Capture Hi-C increases resolution at selected genomic loci [40]. More targeted approaches, including Hi-C coupled with chromatin immunoprecipitation (HiChIP) and chromatin interaction analysis with paired-end tag sequencing (ChIA–PET), combine chromatin interaction mapping with protein-centric enrichment strategies to identify enhancer–promoter contacts associated with specific regulatory factors [42]. These studies have demonstrated that SEs frequently function within three-dimensional regulatory domains, where long-range chromatin interactions contribute to the coordination of gene expression programs [32].

Recent advances in single-cell and multimodal technologies have further refined the investigation of SE biology by enabling the analysis of regulatory landscapes at cellular resolution [48]. Single-cell RNA sequencing (scRNA-seq) provides insights into transcriptional heterogeneity, whereas single-cell assay for transposase-accessible chromatin sequencing (scATAC-seq) enables the characterization of cell-specific chromatin accessibility profiles [23, 49]. The integration of transcriptomic and epigenomic measurements through multi-omics platforms has facilitated the direct linking of chromatin states to gene expression within individual cells. In parallel, spatial transcriptomics preserves tissue architecture while capturing gene expression patterns in situ, providing additional context for the investigation of SE-associated regulatory networks. Collectively, the integration of chromatin profiling, functional interrogation, three-dimensional genome mapping and single-cell multi-omics has established a comprehensive framework for defining SE landscapes and elucidating their regulatory functions in both physiological and pathological settings [50].

#### Experimental models for mechanistic and translational validation

Experimental models are required to determine whether annotated SEs represent functional regulatory dependencies and whether these dependencies persist across biological contexts. Two-dimensional (2D) cultures remain the most accessible platform for chromatin profiling, locus-specific perturbation, and high-throughput drug screening [51, 52] (Fig. 2). They enable controlled comparison of untreated, genetically modified, and therapy-exposed states. However, prolonged culture can select stable subclones, while the absence of tissue architecture, stromal interactions, and physiological gradients limits analysis of microenvironment-dependent or treatment-induced SE remodeling [53]. Patient-derived organoids (PDOs) provide greater biological complexity by preserving aspects of tumor architecture, patient-specific genomic features, and treatment response [53–55]. They are also compatible with longitudinal drug exposure and molecular profiling. Nevertheless, conventional PDOs incompletely reproduce immune, vascular, stromal, and systemic endocrine inputs, and their regulatory states can be influenced by culture media and extracellular matrix [56].

Genetically engineered mouse models (GEMMs) allow SE-associated mechanisms to be examined within intact tissues and defined genetic backgrounds [57–59]. Temporal or lineage-restricted perturbation can distinguish effects on tumor initiation, progression, and therapeutic response. Their principal limitation is species specificity, because enhancer sequences, transcription factor occupancy, and chromatin organization may differ between mouse and human tissues [60]. Patient-derived xenografts (PDXs) complement GEMMs by maintaining human material in vivo and preserving clinically relevant genomic heterogeneity and selected regulatory programs [61–65]. However, conventional PDXs require immunodeficient hosts. Human stromal components are progressively replaced by murine cells, and engraftment may select aggressive subclones, thereby altering tumor-microenvironment interactions and underrepresenting intratumoral diversity [61, 66].

Each model answers a different question. Two-dimensional cultures are well suited to controlled perturbation, PDOs retain more patient-specific architecture, GEMMs allow lineage- and time-restricted studies in intact tissues, and PDXs preserve human tumor material in vivo. A convincing validation strategy should use these systems in a complementary sequence rather than treat any one of them as definitive. Agreement across models strengthens causal inference; disagreement may expose a dependency on species, microenvironment, treatment schedule, or clonal composition [65, 67]. Model origin, passage history, cellular composition, and treatment conditions should therefore accompany every SE analysis.

#### Challenges and limitations in SE identification and annotation

Despite substantial advances in genomic technologies and computational methodologies, the identification and annotation of SEs remain subject to several conceptual and technical challenges. Most current approaches rely on epigenomic features, particularly H3K27ac enrichment, to distinguish SEs from typical enhancers [37]. However, SE annotation is highly dependent on the analytical framework employed. The widely used Rank Ordering of Super-Enhancers (ROSE) algorithm defines SEs by stitching adjacent enhancer elements and ranking them according to signal intensity, making SE calls sensitive to parameters such as stitching distance and threshold selection [8]. Consequently, different analytical settings can generate substantially different SE landscapes, limiting reproducibility and cross-study comparisons. Moreover, enhancer activity frequently exists along a continuum, raising ongoing debate as to whether SEs represent a distinct class of regulatory elements or the upper end of a quantitative enhancer spectrum [20].

The increasing application of single-cell and multimodal technologies has provided new opportunities to investigate SE biology while introducing additional analytical complexity. Single-cell datasets are inherently sparse and susceptible to technical variability, complicating the accurate identification of regulatory elements [47]. In tumor samples, cellular heterogeneity, variable tumor purity and the presence of stromal and immune populations can further confound the interpretation of SE activity [68]. Robust normalization, batch-effect correction and cell-type deconvolution strategies are therefore essential for resolving cell-specific regulatory programs [43].

Additional challenges arise from the integration of diverse omics datasets. Linking chromatin accessibility, histone modifications, chromatin interactions and transcriptional outputs remains computationally demanding and often depends on platform-specific analytical assumptions [69]. Furthermore, SE landscapes are highly dynamic and can be remodeled during development, differentiation and disease progression [70]. Future efforts should focus on establishing standardized criteria for SE annotation, incorporating functional perturbation data into enhancer classification and developing integrative frameworks capable of capturing regulatory dynamics across biological contexts [71]. Such advances will improve the reproducibility and biological interpretability of SE studies and facilitate a more comprehensive understanding of enhancer-mediated gene regulation [20].

## Dynamic regulatory mechanisms governing SE activity

Mapping a candidate SE does not explain how the domain forms, persists, or changes with cell state. Those processes involve transcription factors, chromatin remodelers, three-dimensional genome architecture, non-coding RNAs, and, in some cancers, extrachromosomal DNA. Transcriptional condensates may contribute at selected loci, but their role should be distinguished from the broader effects of cofactor occupancy and chromatin contact. The following sections therefore separate mechanisms tested by perturbation from associations inferred mainly from spatial proximity or chromatin enrichment.

### Pioneer factors, chromatin remodelers, and the epigenetic priming of SEs

**Fig. 3:**
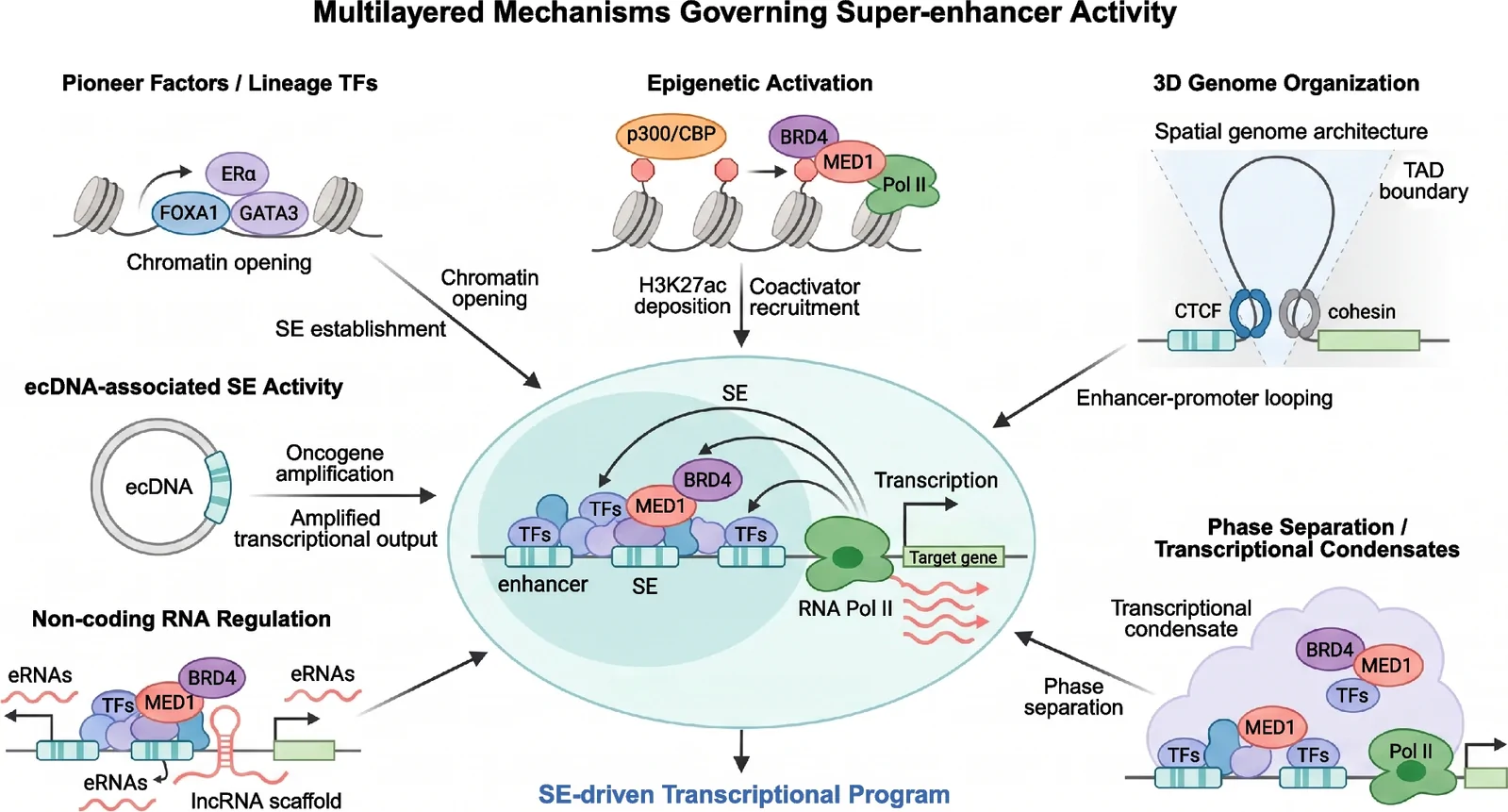
Multilayered regulatory mechanisms governing super-enhancer activity. Pioneer and lineage-determining transcription factors, exemplified by FOXA1, GATA3 and ERα in luminal breast cancer, can promote chromatin opening and super-enhancer (SE) establishment. Epigenetic activation involves p300/CBP-mediated deposition of H3K27ac, accompanied by the recruitment of BRD4, MED1 and RNA polymerase II to active regulatory regions. Three-dimensional genome organization, including topologically associating domains and CTCF–cohesin-associated chromatin looping, helps constrain enhancer-promoter contacts and contributes to regulatory specificity. Multivalent interactions among transcription factors and coactivators, including BRD4 and MED1, may promote biomolecular phase separation and dynamic transcriptional condensates that locally concentrate regulatory components. In selected contexts, enhancer RNAs and long non-coding RNAs contribute to enhancer–promoter communication and regulatory-complex assembly, with some lncRNAs serving as molecular scaffolds. In cancer, extrachromosomal DNA may carry amplified oncogenes together with enhancers or SE-like elements, facilitating regulatory interactions and elevated transcriptional output. The central schematic integrates these mechanisms at an SE-associated regulatory locus, linking chromatin accessibility, coactivator recruitment, spatial organization, and RNA-mediated regulation to target-gene transcription. Arrows indicate conceptual contributions to SE activity rather than a fixed temporal sequence. The contribution of each mechanism varies across cellular states and genomic loci; condensate formation and non-coding RNA dependence are not assumed to be universal requirements for SE function. *Created in BioRender. Miao, H. (2026) *https://BioRender.com/zw0m8xm

The establishment of many SEs involves the coordinated actions of pioneer transcription factors and chromatin-remodeling machinery that collectively convert inaccessible chromatin into transcriptionally permissive regulatory domains [72, 73] (Fig. 3). Pioneer factors can engage nucleosomal DNA within relatively inaccessible chromatin and thereby facilitate the recruitment of additional transcriptional regulators. In several epithelial and hormone-responsive lineages, FOXA1 represents a prototypical pioneer factor that promotes chromatin accessibility by displacing nucleosomes and enabling the binding of lineage-defining transcription factors and coactivator complexes. This process is frequently accompanied by the recruitment of chromatin-remodeling complexes, including switch/sucrose non-fermentable (SWI/SNF), which further reorganize local chromatin architecture and establish an environment conducive to enhancer activation [74, 75].

The formation and stabilization of SEs are reinforced through extensive epigenetic remodeling. Histone acetyltransferases such as p300 and CREB-binding protein (CBP) catalyze the deposition of histone H3 lysine 27 acetylation (H3K27ac), a hallmark of active enhancers that facilitates coactivator recruitment and sustains an open chromatin state [76, 77]. The accumulation of H3K27ac further promotes transcription factor occupancy, generating a feed-forward regulatory circuit that amplifies enhancer activity and contributes to the emergence of SEs [78].

In luminal breast cancer, SE establishment is closely linked to the coordinated activity of estrogen receptor alpha (ERα), FOXA1 and GATA3 [17, 79]. ERα functions as a central transcriptional regulator that recruits coactivators and chromatin modifiers to hormone-responsive regulatory elements, whereas FOXA1 enhances chromatin accessibility and facilitates ERα binding [80, 81]. GATA3 further contributes to the maintenance of luminal transcriptional programs by stabilizing enhancer–promoter interactions and higher-order chromatin organization. Together, these factors form interconnected regulatory networks that sustain lineage identity while promoting oncogenic transcriptional programs. Dysregulation of pioneer factor activity can remodel SE landscapes and redirect transcriptional outputs, thereby contributing to tumor initiation, progression and therapeutic adaptation. These observations illustrate how pioneer factors and epigenetic remodeling contribute to SE architecture and context-specific gene expression programs.

### Spatial genome organization and phase separation in SE function

The regulatory activity of SEs is closely linked to higher-order chromatin organization, which facilitates communication between distal regulatory elements and their target genes. SEs are frequently located within topologically associating domains (TADs), self-interacting chromosomal regions that constrain enhancer–promoter interactions and contribute to regulatory specificity [12]. Architectural proteins, including CCCTC-binding factor (CTCF) and cohesin, play central roles in establishing and maintaining these chromatin domains by mediating long-range looping interactions between SEs and target promoters [82, 83]. Through this three-dimensional organization, SEs can coordinate the expression of lineage-defining and oncogenic genes while minimizing inappropriate regulatory contacts [84].

Disruption of chromatin architecture can profoundly alter SE function. Structural variations, deletions or rearrangements affecting TAD boundaries may generate aberrant enhancer–promoter interactions, a phenomenon often referred to as enhancer hijacking [85, 86]. In T-cell acute lymphoblastic leukemia, for example, disruption of domain boundaries enables ectopic interactions between distal regulatory elements and the MYC locus, resulting in oncogenic transcriptional activation [87, 88]. These observations highlight the importance of chromatin topology in maintaining the fidelity of SE-mediated gene regulation.

In addition to chromatin looping, increasing evidence supports a role for biomolecular phase separation in SE function [89, 90]. SE-associated coactivators, including BRD4 and MED1, contain intrinsically disordered regions that facilitate multivalent molecular interactions and may promote the formation of dynamic transcriptional condensates [13]. These condensates concentrate transcription factors, Mediator complexes and RNA polymerase II within discrete nuclear compartments, thereby enhancing the efficiency of transcriptional initiation and elongation [29]. Unlike static regulatory structures, phase-separated condensates exhibit dynamic assembly and disassembly in response to cellular and environmental cues, providing a mechanism for rapid modulation of gene expression. The functional significance of phase separation is further supported by pharmacological studies demonstrating that bromodomain and extraterminal domain (BET) inhibitors, such as JQ1, disrupt BRD4-associated condensates and preferentially suppress SE-driven transcriptional programs [13]. Conversely, genetic alterations that enhance condensate formation may reinforce aberrant transcriptional activity and contribute to malignant phenotypes. Together, chromatin topology and condensate-like regulatory assemblies provide complementary mechanisms through which SEs coordinate context-dependent transcription in normal physiology and disease.

### Non-coding RNAs in SE-mediated transcriptional regulation

Increasing evidence indicates that non-coding RNAs (ncRNAs) are integral components of SE-mediated regulatory networks, contributing to chromatin organization, transcriptional regulation and cellular state maintenance. Among these, enhancer RNAs (eRNAs) are transient transcripts generated from active SE regions that have emerged as important modulators of enhancer function [14, 91]. Although some eRNAs may reflect enhancer activity, accumulating evidence indicates that selected eRNAs can contribute functionally to enhancer-promoter communication [92]. In selected contexts, interactions between eRNAs and components of the cohesin and Mediator complexes have been reported to reinforce higher-order chromatin architecture, thereby supporting efficient transcriptional activation of SE-associated target genes. In breast cancer, eRNAs derived from ESR1-associated SEs contribute to estrogen-responsive chromatin interactions and sustained transcriptional activity, suggesting a potential contribution to endocrine adaptation in selected luminal breast cancer models [93].

Long non-coding RNAs (lncRNAs) provide an additional layer of SE regulation through their ability to function as molecular scaffolds that coordinate protein complexes and chromatin-modifying enzymes [94]. Accumulation of lncRNAs at regulatory loci can facilitate the assembly of transcriptional machinery, modulate local chromatin states and influence enhancer activity [95]. Some SE-associated lncRNAs have also been shown to regulate distal genomic regions through long-range chromatin interactions [96]. For example, the SE-derived lncRNA CCAT1-L promotes chromatin looping and enhances MYC transcription in colorectal cancer, illustrating how lncRNAs can extend the regulatory influence of SEs beyond their local genomic environment [97, 98]. In addition, interactions between lncRNAs and epigenetic regulators, including Polycomb-associated proteins, contribute to the dynamic remodeling of transcriptional programs in both normal and malignant cells.

Collectively, eRNAs and lncRNAs function as important regulatory components linking chromatin architecture, epigenetic regulation and transcriptional control [99]. Through their ability to coordinate enhancer activity and regulatory complex assembly, ncRNAs contribute to the establishment and maintenance of cell-type-specific gene expression programs [100]. Dysregulation of ncRNA–SE circuits has been implicated in tumor progression, metastasis and therapeutic resistance, suggesting that these regulatory interactions may represent potential targets for therapeutic intervention [101].

### ecDNA-associated SEs and oncogenic transcriptional amplification

Extrachromosomal DNA (ecDNA) has emerged as an important mechanism of oncogene activation in cancer [102, 103]. These circular DNA elements can harbor oncogenes together with enhancer or SE-like regulatory elements, creating highly active regulatory units that drive transcriptional amplification [104]. Unlike chromosomal SEs, which are generally constrained by local chromatin architecture and topological boundaries, ecDNA-associated SEs can engage in extensive regulatory interactions that facilitate high levels of oncogene expression [105]. The clustering of ecDNA molecules within the nucleus further promotes the local accumulation of transcription factors, coactivators and RNA polymerase II, thereby enhancing transcriptional output [106].

The ecDNA–SE axis also contributes to tumor evolution and therapeutic resistance [107, 108]. Dynamic changes in ecDNA copy number enable rapid adaptation to selective pressures and promote transcriptional heterogeneity [109]. Recent studies suggest that ecDNA-driven transcriptional programs exhibit heightened dependence on SE-associated regulatory machinery, which may create sensitivity to BET and (cyclin-dependent kinase 7) CDK7 inhibition in selected models [110]. However, ecDNA amplification and structural reorganization may provide alternative routes to therapeutic escape [111]. These findings identify ecDNA-associated SEs as a distinct mechanism of transcriptional dysregulation and a potential target for therapeutic intervention in cancers characterized by oncogene addiction.

## Biological functions of SEs in development and tissue homeostasis

The mechanisms described above matter biologically only when they alter cell-state-specific transcription. In normal tissues, SE-associated circuits have been linked to developmental transitions, lineage specification, immune responses, and metabolic homeostasis. Their activity changes with developmental stage, cell identity, and environmental input. The examples below distinguish functional evidence from chromatin association, because an SE annotation alone does not establish that a regulatory domain is necessary for a physiological process.

### SEs in lineage specification and cell identity maintenance

**Fig. 4:**
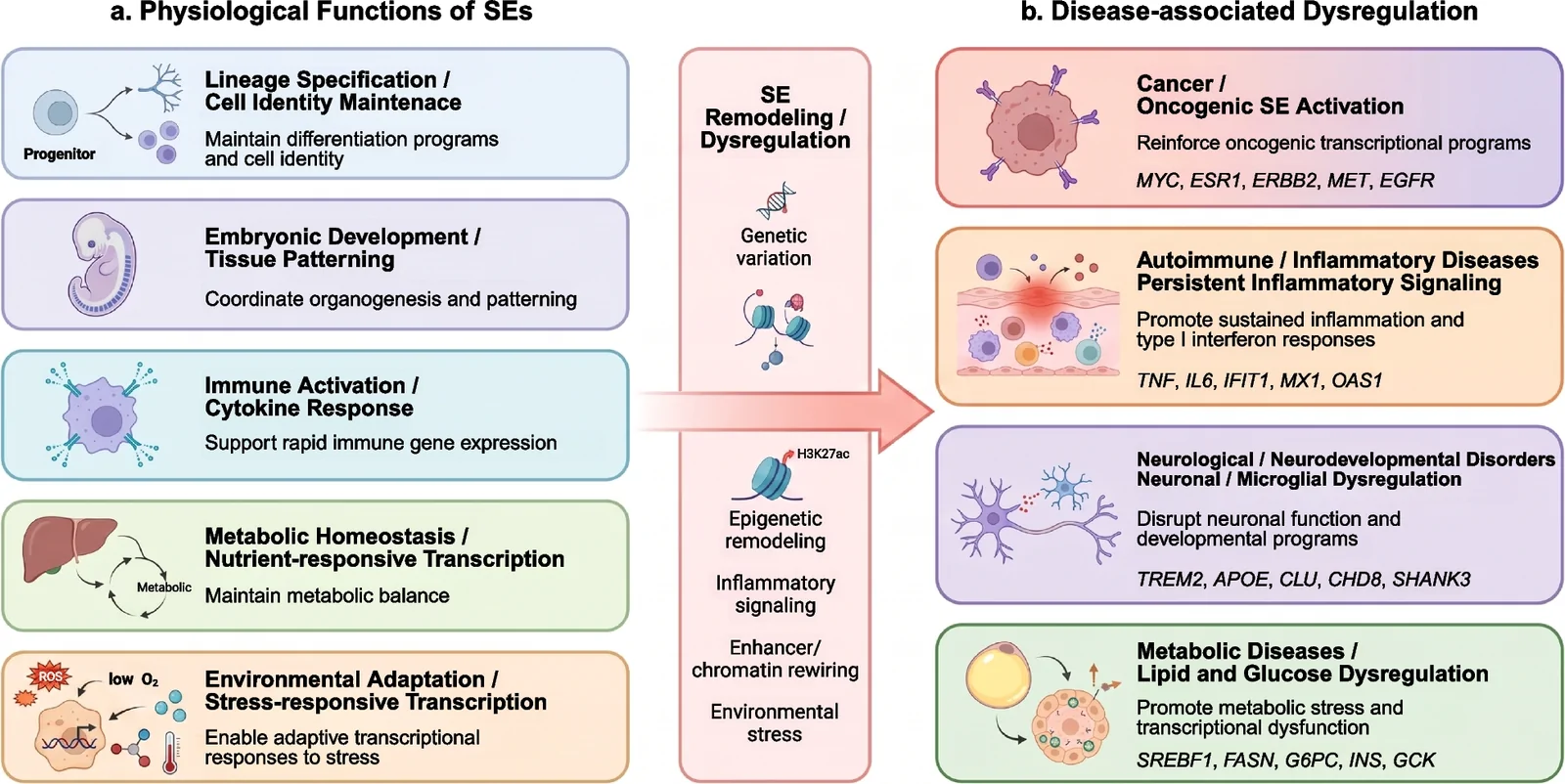
Physiological functions and disease-associated dysregulation of SEs. **a** Physiological roles of super-enhancers (SEs) in coordinating cell identity and adaptive transcriptional programs. SE-associated regulation supports lineage specification and the maintenance of differentiated cell states, as illustrated by progenitor-cell differentiation. During embryonic development, these regulatory programs contribute to organogenesis and tissue patterning. In immune cells, SEs support the induction of immune-response genes following activation and cytokine stimulation. Nutrient-responsive transcription contributes to metabolic homeostasis, whereas stress-responsive regulation enables adaptation to environmental challenges, including oxidative stress and reduced oxygen availability. These functions illustrate how SE-associated transcription integrates developmental, cellular, and environmental signals. **b** Disease-associated alterations in SE regulation across four major disease categories. In cancer, aberrant SE activation can reinforce oncogenic transcriptional programs, illustrated by *MYC*, *ESR1*, *ERBB2*, *MET*, and *EGFR*. In autoimmune and inflammatory diseases, dysregulated enhancer activity may sustain inflammatory signaling and type I interferon responses, represented by *TNF*, *IL6*, *IFIT1*, *MX1*, and *OAS1*. In neurological and neurodevelopmental disorders, altered regulatory programs may affect neuronal function, microglial states, and development, with *TREM2*, *APOE*, *CLU*, *CHD8*, and *SHANK3* shown as representative disease-associated genes. In metabolic diseases, disturbed lipid- and glucose-regulatory programs are illustrated by *SREBF1*, *FASN*, *G6PC*, *INS*, and *GCK*. The central schematic highlights genetic variation, epigenetic remodeling, inflammatory signaling, enhancer and chromatin rewiring, and environmental stress as potential contributors to SE remodeling or dysregulation. The connecting arrow depicts a conceptual relationship between physiological regulation and disease-associated alterations rather than an inevitable progression. The listed genes illustrate the indicated regulatory programs; their association with SEs and their functional dependence on specific regulatory loci require evaluation in the relevant cellular and disease contexts. *Created in BioRender. Miao, H. (2026) *https://BioRender.com/zw0m8xm

SEs are preferentially associated with genes that control cellular identity, lineage commitment and tissue-specific function [112] (Fig. 4). By integrating the activity of lineage-defining transcription factors, coactivators and chromatin regulators, SEs help establish transcriptional programs that support stable cell-state maintenance [113]. These regulatory elements are frequently located near genes encoding key developmental regulators and can occupy important positions within cell-type-specific gene regulatory networks [114].

A characteristic feature of SE-mediated regulation is the formation of interconnected autoregulatory and feed-forward circuits involving lineage-specifying transcription factors [115]. Such networks reinforce transcriptional memory and sustain the expression of identity-defining genes, thereby preserving lineage fidelity during development and tissue homeostasis [116]. In parallel, SEs coordinate chromatin accessibility with context-dependent transcriptional activation, enabling the establishment and maintenance of specialized cellular functions [117].

Disruption of SE activity can alter lineage-specific transcriptional programs and destabilize cellular identity [118]. Aberrant remodeling of SE landscapes has been linked to defective differentiation, inappropriate cell-state transitions and disease-associated transcriptional reprogramming [119, 120]. Together, these observations indicate that SEs contribute to lineage specification, transcriptional stability and tissue homeostasis across diverse biological contexts.

### SEs in embryonic development and tissue patterning

Building on their roles in lineage specification, SE landscapes can be dynamically remodeled as embryonic cells transition between developmental states. By concentrating lineage-determining transcription factors and transcriptional coactivators at key developmental loci, SEs support high-level and context-dependent expression of genes involved in germ-layer specification, morphogenesis and tissue patterning [121, 122].

During early Xenopus development, the maternal transcription factors Foxi2 and Sox3 bind cis-regulatory modules before gastrulation and subsequently facilitate EP300 recruitment and H3K27ac deposition. These regulatory modules are preferentially enriched within ectoderm-specific SEs, where they promote robust activation of ectodermal gene-expression programs during germ-layer specification [123]. In mouse cranial neural crest cells, a distal regulatory region containing multiple SEs establishes long-range inter-TAD interactions with the Hoxa cluster. Two components of this region, HIRE1 and HIRE2, regulate Hoxa2 and Hoxa3 expression in spatially defined cranial neural crest populations, thereby contributing to pharyngeal-arch and craniofacial patterning [124].

SE-associated regulation also contributes to organogenesis. During mouse cardiac development, the *Upperhand* (*Uph*) lncRNA locus overlaps the *Hand2*-associated SE. Transcription through this locus, rather than the mature* Uph* transcript itself, was reported to maintain active enhancer chromatin and RNA polymerase II occupancy, thereby supporting *Hand2* expression. Premature termination of *Uph* transcription reduced *Hand2* expression and caused right-ventricular hypoplasia and embryonic lethality, although subsequent studies suggested additional contributions from local DNA regulatory elements [125]. In mouse trophoblast stem cells, SE profiling identified a core regulatory network involving CDX2, GATA3 and TEAD4, which is dynamically remodeled during trophoblast differentiation and placental lineage specification [9].

These developmental examples do not support SEs as autonomous timing devices. Instead, they show that specific enhancer assemblies coordinate transcription-factor occupancy, active chromatin, and long-range contacts at defined loci and developmental stages. Disruption of these regulatory architectures can impair lineage specification and tissue morphogenesis, providing a mechanistic link between SE dysfunction and congenital developmental abnormalities.

### SEs in immune cell activation and functional plasticity

In the immune system, SEs regulate both activation dynamics and functional plasticity [126]. Following T cell receptor (TCR) stimulation, cytokine-associated SEs at loci such as IL2 and IFNG acquire increased H3K27ac, supporting rapid transcriptional activation during T cell effector responses [87, 127]. This response is associated with the recruitment of stimulus-responsive transcription factors, including nuclear factor kappa B (NF-κB), activator protein 1 (AP-1) and nuclear factor of activated T cells (NFAT) [128].

In regulatory T cells (Tregs), SEs maintain lineage-defining FOXP3 expression, whereas these regulatory elements are less active or epigenetically constrained in conventional effector T cells, supporting lineage segregation within CD4 + T cell subsets [129, 130]. This differential SE activity contributes to the establishment and maintenance of immune cell identity [131].

Immune-cell plasticity also involves stimulus-dependent enhancer remodeling. LPS exposure increases activity at pro-inflammatory loci such as TNF and IL6 in macrophage models [132], whereas IL-4 promotes regulatory regions associated with tissue repair, including Arg1 and Mrc1 [133]. Under chronic inflammatory conditions, SE configurations can become stably biased toward pro-inflammatory states, contributing to sustained immune activation and disease persistence [134]. These examples show how enhancer use follows cellular state, but the extent to which each remodeled domain meets functional SE criteria must be established separately [135].

### SEs in metabolic homeostasis and environmental adaptation

SEs contribute to metabolic homeostasis by integrating nutrient, hormonal and environmental cues with tissue-specific transcriptional programs [11, 136]. In pancreatic β-cells, glucose-responsive SEs at the insulin locus are occupied by lineage-defining transcription factors, including PDX1 and MAFA, thereby supporting insulin expression and secretion in response to elevated glucose levels [137, 138]. In hepatocytes, fasting-associated SEs at gluconeogenic genes such as G6PC and PCK1 are activated through CREB- and FOXO1-dependent pathways, promoting hepatic glucose production during nutrient deprivation [139].

Environmental stress can reshape metabolic enhancer programs. Cold exposure activates β-adrenergic signaling in brown adipose tissue and increases activity at thermogenic loci, including UCP1 and PGC1α, through PRDM16-associated complexes [140, 141]. This response supports heat production and energy expenditure [142]. Chronic overnutrition, by contrast, can increase enhancer activity at inflammatory loci such as CCL2 and IL1β in adipose tissue [143]. Such changes may contribute to low-grade inflammation, insulin resistance and metabolic dysfunction. SE-associated regulation thus connects environmental signals with tissue-specific transcription, but causality must be evaluated at individual loci [144].

## SE dysregulation in human diseases

The regulatory properties that support normal cell identity and physiological adaptation can become pathogenic when they are redirected by genetic lesions, altered signaling, or epigenetic dysregulation. Disease-associated SEs may activate aberrant transcriptional programs, reinforce maladaptive cell states, or increase dependence on specific regulatory factors. These effects remain strongly context dependent and should not be inferred solely from elevated H3K27ac or cofactor occupancy. This section examines the mechanisms and evidence underlying SE dysregulation in cancer and in immune, neurological, and metabolic diseases.

### General principles of oncogenic SE activation and rewiring

Oncogenic SE states can emerge through transcription-factor redistribution, epigenetic remodeling, altered three-dimensional genome organization, and genetic alterations. Lineage-defining or oncogenic transcription factors may occupy pre-existing enhancers, recruit coactivators, and strengthen or assemble aberrant regulatory domains [15, 33, 100]. Oncogenic factors and altered architectural proteins can also reorganize enhancer–promoter contacts, thereby redirecting regulatory activity toward growth-promoting genes [84, 90]. Genetic lesions provide a distinct route. Structural variants can juxtapose active enhancers with proto-oncogenes, whereas focal or extrachromosomal amplification can co-amplify oncogenes and their regulatory elements [85, 145, 146]. On ecDNA, intermolecular enhancer–gene interactions within nuclear hubs may further increase oncogene transcription [104, 105]. Enhancer hijacking or co-amplification, however, should not automatically be equated with SE formation; this classification requires locus-resolved evidence of clustered regulatory activity. Although mechanistically distinct, these processes can converge on sustained or inducible expression of genes that promote proliferation, survival, and lineage plasticity.

Oncogenic SE states are further shaped by lineage and environmental context. Extrinsic and oncogenic signaling can alter transcription-factor occupancy, cofactor recruitment, and chromatin accessibility without changing the underlying DNA sequence [33, 100, 147]. These inputs may activate previously inactive elements, strengthen existing enhancer clusters, or redirect regulatory contacts toward alternative target genes. Multivalent coactivator interactions and aberrant chromatin looping can reinforce selected oncogenic programs [13, 29, 90]. However, condensate-like behavior and strong H3K27ac enrichment are neither universal features nor sufficient evidence of causal dependency. The functional consequence of an annotated SE depends on promoter compatibility, three-dimensional topology, and the requirement for its target gene within the relevant tumor lineage.

This distinction is essential when interpreting cancer epigenomes. A tumor-enriched SE may reflect oncogenic transcription, copy-number alteration, or differences in cellular composition without constituting a selective vulnerability. Establishing causality requires copy-number-aware chromatin analysis, locus-resolved target-gene assignment, and endogenous perturbation of the regulatory element, ideally followed by phenotypic rescue [8, 20, 32, 146]. Validation across cell lines, patient-derived models, and normal cellular counterparts is also needed to determine the persistence and selectivity of the dependency. Breast cancer provides an informative setting because its molecular subtypes couple distinct lineage and receptor-signaling programs to different enhancer landscapes. Functional evidence is comparatively strong for selected ERα-associated and triple-negative breast cancer circuits, whereas proposed HER2-associated SE dependencies remain less comprehensively validated [16–18, 147–149]. The following sections therefore use breast cancer to compare established regulatory dependencies with candidate associations for which causal evidence remains incomplete.

### Breast cancer as a model of subtype-specific SE regulation

Breast cancer is examined in depth here because its major molecular subtypes provide a comparatively well-defined system for relating lineage factors, hormone-receptor and receptor-tyrosine-kinase signaling, tumor microenvironmental cues and treatment adaptation to distinct regulatory states [148]. Functional perturbation and multi-omic studies have identified selected SE dependencies in luminal and triple-negative models, while the evidence for other proposed circuits remains more limited. Breast cancer therefore provides a useful framework for comparing the strength and context dependence of SE evidence across luminal, HER2-positive and triple-negative disease [16, 17, 147, 149].

#### ERα-associated enhancer and SE circuits in luminal breast cancer

**Fig. 5:**
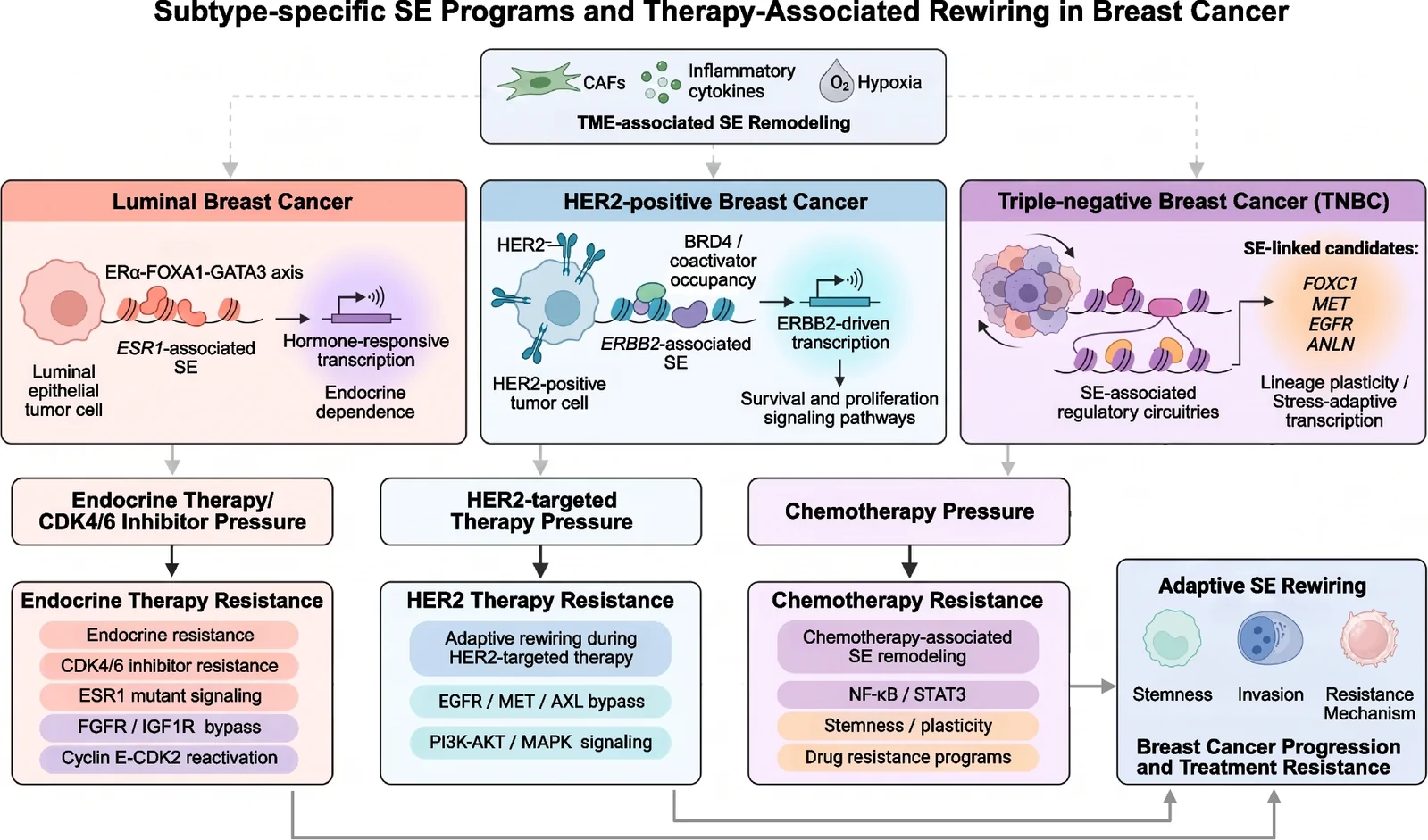
Subtype-specific SE programs and therapy-associated rewiring in breast cancer. Breast cancer subtypes exhibit distinct enhancer and super-enhancer (SE)-associated regulatory landscapes. Luminal tumors show ERα–FOXA1–GATA3 and ESR1-associated programs, whereas HER2-positive tumors are represented by ERBB2-associated regulatory activity. Triple-negative breast cancer exhibits heterogeneous circuits, with FOXC1, MET, EGFR, and ANLN shown as illustrative SE-associated genes. Cancer-associated fibroblasts, inflammatory cytokines, and hypoxia may further influence these landscapes. Treatment-associated adaptations include ESR1 mutation-associated endocrine resistance, FGFR/IGF1R bypass, and cyclin E–CDK2 reactivation in endocrine/CDK4/6 inhibitor-resistant settings; EGFR/MET/AXL bypass and PI3K–AKT/MAPK signaling during HER2-targeted treatment; and NF-κB/STAT3-associated stemness and plasticity during chemotherapy. These examples illustrate resistance-associated programs that may intersect with enhancer remodeling, rather than uniformly established consequences of SE rewiring. Converging arrows summarize potential links to invasion, progression, and resistance. Treatment-induced enhancer changes can also support differentiation or other cellular responses. Distinguishing causal SE remodeling from broader stress responses and selection of preexisting states requires temporally resolved profiling and locus-specific perturbation. The illustrated mechanisms are context dependent and do not necessarily coexist in individual tumors. *Created in BioRender. Miao, H. (2026) *https://BioRender.com/zw0m8xm

In luminal breast cancer, estrogen receptor alpha (ERα), encoded by ESR1, operates within a lineage-specific regulatory network comprising pioneer factors, coactivators, and distal regulatory elements. Following estrogen stimulation, ERα is recruited to a subset of accessible chromatin, with its occupancy shaped by local DNA sequence, cooperating transcription factors, and chromatin context [80, 150]. This regulatory landscape is not invariant. Profiling of primary breast tumors revealed substantial interpatient heterogeneity in ERα binding, whereas the most recurrent sites exhibited stronger enhancer activity and enrichment for breast cancer risk variants [151] (Fig. 5). Conserved principles of ERα regulation therefore coexist with patient-specific enhancer configurations, and SE maps from a single cell line cannot be assumed to represent all luminal tumors.

The BRD4–ERα–RET circuit provides a functionally characterized example. Estrogen-induced BRD4 occupancy at ERα-associated SEs activates a group of estrogen-responsive genes, including RET. RET signaling, in turn, enhances ERα phosphorylation and transcriptional activity, establishing a positive feedback loop in selected ER-positive models [17]. Estrogen stimulation also induces transcription at numerous ERα-bound enhancers [150]. However, eRNA detection primarily indicates regulatory activity rather than functional necessity. At selected loci, eRNA depletion impairs enhancer–promoter communication and estrogen-responsive target-gene activation, supporting locus-specific functions for these transcripts [152]. Similarly, ERα occupancy, H3K27ac enrichment, and eRNA abundance alone cannot establish SE dependency or identify the direct target promoter. Endogenous perturbation of individual constituents, combined with transcriptional and chromatin-contact analyses, is required to demonstrate causality [20, 32]. Luminal breast cancer thus illustrates how hormone-responsive enhancers integrate lineage identity with reversible signal-dependent transcription, while emphasizing that candidate SE dependencies must be resolved at the locus level and validated across clinically relevant models.

#### ERBB2-associated enhancer regulation in HER2-positive breast cancer

HER2-positive breast cancers commonly exhibit ERBB2 amplification and high HER2 expression, but the structure of the 17q12 amplicon and its transcriptional output vary across tumors [153]. Regulatory elements have been identified within and around the ERBB2 locus. CRISPR-mediated deletion of Her2-Enhancer1, a candidate expressed enhancer located within the ERBB2 gene body, altered HER2 expression and downstream signaling in breast cancer cells [18]. These findings support enhancer-mediated regulation of ERBB2 but do not establish a uniformly present or functionally required ERBB2-associated SE. Moreover, because Her2-Enhancer1 is intragenic, deletion-based experiments may confound enhancer activity with disruption of local gene structure. Orthogonal approaches that preserve the underlying DNA sequence are therefore needed. ERBB2 amplification alone is also insufficient to establish SE status because increased DNA abundance can elevate aggregate chromatin-profiling signals. Copy-number-aware normalization is required before H3K27ac or cofactor enrichment is interpreted as regulatory activity.

A rigorous model of ERBB2 regulation therefore requires several layers of evidence. Chromatin profiling should define the active elements and their boundaries, whereas three-dimensional contact data should establish physical linkage to the ERBB2 promoter. Endogenous CRISPR interference, epigenome editing, and combinatorial perturbation are needed to test the necessity of individual elements and the broader regulatory domain [8, 20, 32]. These analyses should include multiple HER2-positive models with different amplicon architectures, hormone-receptor states, and genomic backgrounds [153], together with nonmalignant mammary controls. Current evidence therefore more firmly establishes ERBB2 as an amplification-associated oncogene subject to enhancer regulation than as a universally SE-dependent locus. Therapy-induced regulatory adaptation is considered separately in Section “Adaptive SE rewiring during HER2-targeted therapy”.

#### Subtype- and cell-state-specific SE circuitries in triple-negative breast cancer

Triple-negative breast cancer (TNBC) comprises molecularly and clinically heterogeneous tumors [148]. An initial transcriptomic classification identified six TNBC subtypes [154], which was subsequently refined to the TNBCtype −4 framework—basal-like 1 (BL1), basal-like 2 (BL2), mesenchymal (M) and luminal androgen receptor (LAR)—after immune and stromal signals were recognized as major contributors to the previously defined immunomodulatory and mesenchymal stem-like groups [155]. These tumor-intrinsic categories differ in cell-cycle activity, DNA-damage responses, growth-factor signaling, mesenchymal programs and androgen receptor dependence [155]. However, subtype assignments can vary with cohort composition, sampling strategy and analytical framework [148, 155]. Mapping these transcriptomic categories onto reproducible SE landscapes remains incomplete. Available multi-omic studies instead indicate substantial intertumoral and cell-state-associated SE heterogeneity rather than a single regulatory program shared across TNBC [16, 149].

The strongest functional evidence comes from integrated H3K27ac and transcriptomic profiling combined with CRISPR-mediated perturbation, which identified FOXC1 and MET as genes regulated by TNBC-specific SEs and provided additional evidence for SE-associated regulation of ANLN [16]. Subsequent studies have linked TWEAK–Fn14-dependent SE reprogramming to metabolic and metastatic phenotypes [147]. Complementary CRISPR screens of transcribed SEs have identified regulatory elements required for tumor progression in selected TNBC models [19]. These findings support specific, model-dependent SE dependencies but do not establish uniform dependence across BL1, BL2, M and LAR tumors or across patient populations. Immune-associated SE signals should likewise be interpreted cautiously because bulk profiling may conflate tumor-cell-intrinsic enhancer activity with signals contributed by infiltrating immune and stromal cells [155]. TNBC should therefore be viewed as comprising multiple, context-dependent SE circuitries whose therapeutic relevance requires subtype-resolved profiling, locus-specific perturbation and validation in clinically annotated cohorts.

#### Tumor microenvironment-associated enhancer remodeling and candidate SE regulation in breast cancer

The tumor microenvironment can alter enhancer-associated transcription through paracrine signaling, metabolic stress and spatially restricted cellular interactions [156]. In breast cancer stroma, JUN-dependent enhancer reprogramming has been shown to maintain cancer-associated fibroblast (CAF) activation and promote tumor-cell invasion through non-cell-autonomous mechanisms [157]. Other studies implicate FGFR signaling and fibroblast-derived immunoregulatory programs in shaping tumor–stromal interactions [158, 159], but these observations do not by themselves establish SE formation or a tumor-cell-intrinsic SE dependency. Pro-inflammatory signaling has been linked to activation of a CD47-associated SE in breast cancer cells [160], providing locus-specific evidence that extracellular signals can engage SE-associated regulation. However, this finding does not establish that CAF-derived cytokines, including IL-6, broadly create or maintain functionally required SEs across breast cancer subtypes.

Hypoxia can alter hypoxia-inducible factor (HIF)-dependent transcription, chromatin accessibility and enhancer–promoter communication. In breast cancer cells, hypoxia-induced chromatin interactions at the MALAT1 locus provide direct evidence for locus-specific enhancer rewiring [161]. By contrast, current evidence does not establish genome-wide or functionally required HIF-dependent SE reconfiguration in primary breast tumors. HIF-responsive gene expression or changes in immune-checkpoint expression should therefore not be interpreted as evidence of SE dependency in the absence of chromatin-level and perturbation-based validation. Overall, available data more securely support microenvironment-dependent enhancer remodeling than stable SE reprogramming. Establishing the latter will require cell-type-resolved chromatin profiling, longitudinal sampling and locus-specific perturbation in patient-derived models.

### SE dysregulation in other solid tumors and hematologic malignancies

SE dysregulation is not restricted to breast cancer. In solid tumors, recurrent mechanisms include enhancer hijacking, ecDNA-associated regulatory amplification and lineage-specific transcriptional addiction. Examples discussed in mechanistic studies include MYC regulation by the SE-derived lncRNA CCAT1-L in colorectal cancer, LSD1-dependent SE programs in castration-resistant prostate cancer, ALDH1A1-associated SE activity in ovarian cancer and MYCN amplicon architecture in neuroblastoma [97, 98, 145, 162, 163]. These examples illustrate distinct modes of SE-associated regulation rather than a single pan-cancer mechanism.

Hematologic malignancies provide additional evidence for lineage- and oncogene-centered SE dependencies. T-cell acute lymphoblastic leukemia can acquire ectopic MYC activation through altered regulatory-domain architecture [87], whereas SE-driven TOX2 regulation has been reported in natural killer/T-cell lymphoma [164]. BET, CDK9 and related transcriptional regulators have also been evaluated in lymphoma and leukemia contexts [165–169]. Across both solid and hematologic malignancies, therapeutic interpretation should remain contingent on endogenous locus perturbation and context-specific validation rather than SE annotation alone.

### Enhancer and SE-associated dysregulation in autoimmune and inflammatory diseases

Disease-associated enhancer remodeling, including changes at regions operationally annotated as SEs, has been reported in autoimmune and inflammatory diseases [170]. In rheumatoid arthritis (RA), synovial fibroblasts exhibit active enhancer landscapes at inflammatory and tissue-remodeling loci, including genes encoding cytokines, chemokines and osteoclastogenic factors [171–174]. SE-associated regulatory regions near TNF, IL6, IL1B and TNFSF11, which encodes receptor activator of nuclear factor-κB ligand (RANKL), may integrate NF-κB and AP-1 signaling, thereby supporting persistent inflammatory gene expression, synovial activation and bone-destructive processes [175]. Enhancer RNAs transcribed from these regions may further contribute to enhancer–promoter communication, although their functional contribution requires context-specific validation. Preclinical studies suggest that perturbing SE-associated transcriptional regulators or selected enhancer-derived RNAs can attenuate inflammatory outputs, but therapeutic translation remains at an early stage.

In systemic lupus erythematosus (SLE), enhancer and putative SE remodeling has been associated with the exaggerated type I interferon response that characterizes many patients [176, 177]. Plasmacytoid dendritic cells and other immune populations can exhibit increased enhancer activity at interferon-stimulated genes, including IFIT1, MX1 and OAS1. Moreover, genetic risk variants associated with SLE and other autoimmune diseases are frequently enriched within non-coding regulatory regions, including SE-like elements, where they may alter transcription factor binding and chromatin activity [178, 179]. BET inhibition has been shown in experimental models to dampen inflammatory and interferon-associated transcriptional programs, providing proof of principle that SE-associated regulatory dependencies may be therapeutically tractable. However, given the broad role of SEs in normal immune activation and tolerance, future studies will need to define disease-specific regulatory circuits, cell-type-selective vulnerabilities and therapeutic windows before SE-directed approaches can be advanced for autoimmune disease treatment.

### SE dysregulation in neurological and neurodevelopmental disorders

SE dysregulation has been implicated in neurological and neurodevelopmental disorders through altered control of neuronal, glial and immune-related transcriptional programs. In Alzheimer’s disease (AD), disease-associated enhancer remodeling has been observed in microglia, particularly at loci involved in neuroinflammation, lipid metabolism and phagocytic responses [180, 181]. Regulatory regions near genes such as TREM2, APOE and CLU may acquire increased H3K27ac during disease progression, suggesting that altered SE activity may contribute to sustained inflammatory and stress-response states in the aging brain. Single-nucleus chromatin accessibility analyses further indicate that AD-associated non-coding risk variants, including variants near BIN1 and PICALM, are enriched in microglial regulatory elements, supporting a link between inherited genetic risk and cell-type-specific enhancer function [182, 183]. BET inhibition has shown anti-inflammatory effects in experimental models, but whether these effects are mediated directly through disease-relevant SEs requires further mechanistic validation.

In neurodevelopmental disorders, including autism spectrum disorder (ASD), genetic and epigenetic disruption of SE-associated regulatory networks may impair neuronal differentiation, synaptic maturation and activity-dependent gene expression [184, 185]. De novo mutations and non-coding risk variants can affect genes or regulatory elements associated with synaptic function, chromatin remodeling and neuronal lineage specification, including SHANK3, NRXN1, CHD8 and ARID1B [186]. Variants within neuronal SEs may alter transcription factor occupancy, including factors such as MEF2C and TBR1, thereby perturbing developmental gene regulatory programs [187]. CRISPR-based epigenetic editing has been explored in patient-derived cellular models to modulate disease-associated regulatory elements, but these approaches remain preclinical. Overall, SE-centered analyses provide a useful framework for linking non-coding genetic variation, cell-type-specific chromatin states and disease-associated transcriptional dysregulation in the nervous system.

### SE dysregulation in metabolic diseases

SE dysregulation has been implicated in metabolic diseases through altered transcriptional programs that control lipid metabolism, glucose homeostasis, inflammation and cellular stress responses [188]. In non-alcoholic fatty liver disease (NAFLD), hepatocyte enhancer landscapes are remodeled at genes involved in de novo lipogenesis, fatty acid metabolism and gluconeogenesis, including SREBF1, FASN, SCD1, PCK1 and G6PC [189, 190]. These regulatory changes can be influenced by nutrient-responsive transcription factors such as carbohydrate-responsive element-binding protein (ChREBP) and sterol regulatory element-binding protein 1c (SREBP1c), and may contribute to hepatic lipid accumulation and insulin resistance [191]. Enhancer RNAs derived from metabolic regulatory regions may further modulate enhancer–promoter communication, although their functional relevance should be defined in a cell-type- and disease-stage-specific manner. In parallel, Kupffer cells and other liver-resident immune populations can exhibit inflammatory enhancer activation, with increased chemokine and cytokine expression at loci such as CCL2 and IL1B. These immune regulatory programs are likely to participate in the inflammatory progression from steatosis to non-alcoholic steatohepatitis (NASH) [192].

In type 2 diabetes (T2D), pancreatic β-cell dysfunction is associated with disruption of regulatory elements controlling insulin production, glucose sensing and stimulus–secretion coupling, including loci such as INS, GCK and KCNJ11 [193]. Chronic glucotoxicity, lipotoxicity, oxidative stress and inflammatory cytokines may weaken β-cell identity-associated enhancer programs while increasing regulatory activity at stress-response and pro-apoptotic genes, including BAX and BID. In selected preclinical models, histone deacetylase or BET inhibition influences β-cell survival and inflammatory responses, but tissue specificity, long-term safety, and metabolic effects remain unresolved. SE-centered analyses can therefore help relate metabolic stress to cell-type-specific chromatin remodeling and transcriptional dysfunction in NAFLD, NASH, and type 2 diabetes [194, 195], although individual dependencies require direct testing.

## Therapeutic targeting of SEs and resistance-associated reprogramming

The identification of disease-associated SE programs raises the possibility of therapeutically disrupting regulatory dependencies that sustain pathological cell states. Such dependencies can be targeted indirectly through transcriptional and chromatin regulators or more selectively through perturbation of individual enhancers, enhancer RNAs, or associated signaling circuits. However, an annotated SE is not necessarily a tractable or selective therapeutic target. This section evaluates current targeting strategies, the evidence supporting their mechanisms, and the adaptive SE reprogramming that can limit treatment durability and promote resistance.

### Small-molecule inhibitors targeting SE-associated transcriptional machinery

**Fig. 6:**
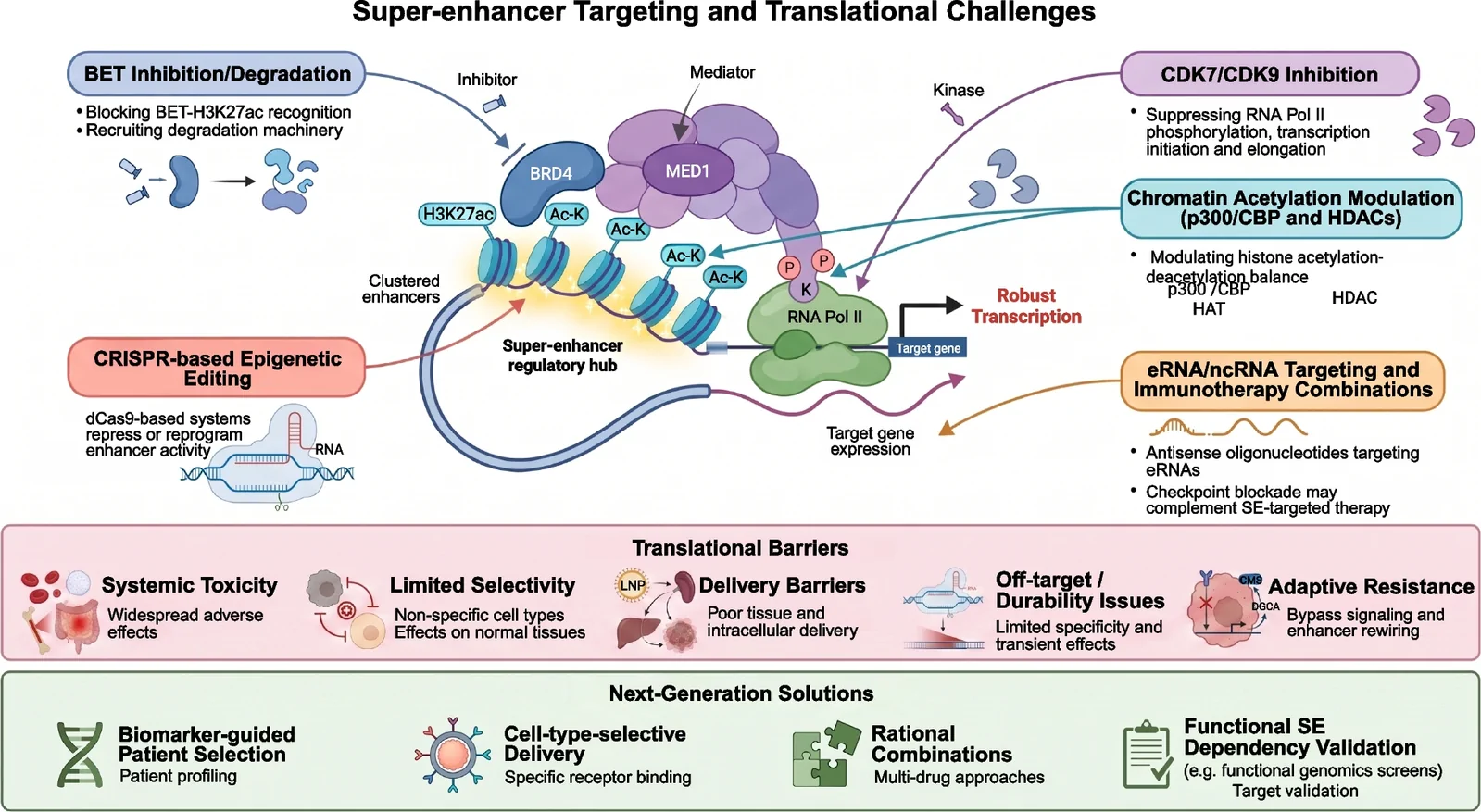
Therapeutic targeting of SE-associated regulation and translational challenges. The central schematic depicts a super-enhancer (SE)-associated regulatory hub comprising acetylated chromatin, BRD4, MED1, and RNA polymerase II. Strategies include bromodomain and extraterminal domain (BET) inhibition or degradation, CDK7 or CDK9 inhibition, and modulation of acetylation through p300/CBP or histone deacetylases. CDK7 and CDK9 regulate distinct, interconnected steps in transcription initiation and productive elongation. Acetylation-directed interventions can have different effects depending on the target and cellular context. Additional approaches include dCas9-based epigenetic editing, antisense oligonucleotides targeting enhancer RNAs, and combinations with immune checkpoint blockade. These interventions may alter SE-associated transcription, although most do not selectively target individual SEs. The middle section summarizes systemic toxicity, limited selectivity, inadequate delivery, off-target or transient effects, and adaptive resistance. The bottom section highlights biomarker-guided selection, cell-type-selective delivery, rational combinations, and functional validation of SE dependencies. Clinical maturity varies across strategies: some transcriptional inhibitors have entered clinical trials, whereas locus-specific editing and enhancer RNA-directed approaches remain largely preclinical. Therapeutic benefits require validation in the relevant disease context. *Created in BioRender. Miao, H. (2026) *https://BioRender.com/zw0m8xm

Therapeutic targeting of SEs is based on the observation that SE-associated genes can show increased sensitivity to perturbation of transcriptional cofactors and elongation machinery. Small-molecule strategies have therefore focused on SE-associated transcriptional regulators and chromatin-modifying enzymes, including BET family members, CDK7, CDK9 and histone deacetylases [196] (Fig. 6). BET inhibitors, including the tool compound JQ1 and related clinical-stage agents, can limit BET protein binding to acetylated chromatin and thereby reduce SE-associated transcriptional activity [197] (Table 2). CDK7 and CDK9 inhibitors interfere with RNA polymerase II phosphorylation and transcriptional initiation or elongation, which can preferentially affect genes driven by high-density regulatory elements [215].

**Table 2:** Therapeutic strategies for modulating super-enhancer-associated transcriptional dependencies

Strategy	Representative agents or platforms	SE-related rationale	Application context	Representative development stage	Principal limitations
BET inhibition	JQ1 (tool compound)	Competes for acetylated-chromatin binding; may attenuate BRD4-dependent SE transcription	BET-dependent cancers; inflammatory programs	Preclinical research tool [26]	Broad transcriptional effects; hematologic/GI toxicity; adaptive resistance
	TQB3617			Phase I monotherapy in relapsed/refractory lymphoma (NCT05110807) [166]	
	NHWD-870 HCl			Phase I published; phase II registered in advanced NUT carcinoma (NCT06527300) [167]	
	Pelabresib			Phase III with ruxolitinib in JAK inhibitor-naive myelofibrosis (MANIFEST-2; NCT04603495) [168]	
BET degradation	ARV-825	BET depletion may be deeper or more sustained than occupancy-based inhibition	BET-dependent cancers; resistance-associated states	Preclinical cancer models [169]	Delivery; pharmacokinetics; tissue selectivity; ubiquitin–proteasome resistance
	MZ1			Preclinical BRD4 degradation tool [198]	
CDK7 inhibition	Samuraciclib	Reduces Pol II phosphorylation and transcriptional initiation	Transcriptionally addicted tumors; candidate SE dependencies	Phase I published [199]; randomized phase II with fulvestrant completed (SUMIT-BC; NCT05963984)	Broad transcription/cell-cycle effects; dose-limiting toxicity; uncertain biomarkers
	THZ1 (tool compound)			Preclinical research tool [200]	
CDK9 inhibition	KB-0742	Restricts Pol II pause release and elongation	Selected MYC- or SE-associated programs	Phase I dose-escalation/expansion published; trial terminated in late 2024 (NCT04718675) [201]	Narrow therapeutic window; myelosuppression; normal-cell transcription
HDAC inhibition	Vorinostat	Alters histone/non-histone acetylation; may remodel enhancer accessibility and reader dependence	Region-specific cancer approvals; investigational SE-directed use	FDA-approved: CTCL [202]	Limited locus specificity; pleiotropy; toxicity; uncertain individual-SE effects
	Romidepsin			FDA-approved: CTCL; US PTCL indication withdrawn [202]	
	Belinostat			FDA accelerated approval: relapsed/refractory PTCL [202]	
	Panobinostat			EU-authorized with bortezomib/dexamethasone: previously treated multiple myeloma; US approval withdrawn in 2022 [202]	
	Entinostat			China-approved with an aromatase inhibitor: HR+/HER2 − advanced breast cancer	
RNA/eRNA targeting	Antisense oligonucleotides	Depletes selected RNAs that may support enhancer–promoter communication or transcription [14, 19, 99]	Validated RNA-dependent enhancer circuits	Preclinical SE/eRNA targeting	Target validation; RNA stability; delivery; context-dependent function
	siRNA			Preclinical SE-associated regulatory RNA targeting	
	Cas13			Preclinical SE/eRNA-targeting platform	
CRISPR-based epigenetic editing	dCas9-KRAB	Locus-specific chromatin modification can repress or activate candidate SEs [20, 203, 204]	Functional validation; precision regulatory modulation	Preclinical SE modulation; functional research platform	Delivery; cell specificity; durability; immunogenicity; off-target chromatin effects
	dCas9-p300/CBP			Preclinical SE modulation; functional research platform	
	dCas9-DNMT3A			Preclinical SE modulation; DNA methylation platform	
	dCas9-LSD1			Preclinical SE modulation; enhancer perturbation platform	
Targeted delivery	Lipid nanoparticles	Delivers RNA, guide RNA, or genome/epigenome editors	Enables nucleic-acid/CRISPR-based SE modulation	Clinical validation for selected non-SE cargos; SE-modulating payload delivery is preclinical [205, 206]	Biodistribution; penetration; endosomal escape; immunogenicity; capacity; scalability
	Engineered virus-like particles			Preclinical genome/epigenome editor delivery [207, 208]	
Transcriptional-regulator combinations	BET plus CDK9 inhibition	Perturbs complementary transcriptional mechanisms; may limit compensatory adaptation	Biomarker-defined combined dependencies	Preclinical: JQ1 plus CDKI-73 [209]	Overlapping toxicity; schedule optimization; patient selection
	BET targeting plus HDAC inhibition			Preclinical: ARV-771 plus romidepsin [210]	
	BET plus ENL inhibition			Preclinical combination studies [197]	
Immunotherapy combinations	BET-targeting agent plus PD-1/PD-L1 blockade	May alter checkpoint, antigen-presentation, or T-cell dysfunction programs	Selected tumor–immune regulatory dependencies	Preclinical representative SE-related context [211, 212]	Context specificity; immune/overlapping toxicity; normal immune disruption
	Entinostat plus pembrolizumab			Phase II PEMDAC: metastatic uveal melanoma [213]	
	Entinostat plus pembrolizumab			Phase Ib: MDS/AML after hypomethylating-agent failure [214]	

Representative stages are based on information available by October 2026 and may differ by indication. Trial phase, completion/termination, and marketing authorization are distinct. Approvals are jurisdiction- and indication-specific; neither approval nor trial participation establishes SE-specific efficacy. All listed HDAC agents, RNA platforms, and delivery systems remain investigational for SE-directed use. Entinostat–pembrolizumab trials were not validated as SE-directed therapy

*Abbreviations*: *AML* acute myeloid leukemia, *BET* bromodomain and extraterminal domain, *CBP* CREB-binding protein, *CDK* cyclin-dependent kinase, *CRISPR* clustered regularly interspaced short palindromic repeats, *CTCL* cutaneous T-cell lymphoma, *dCas9* catalytically inactive Cas9, *DNMT3A* DNA methyltransferase 3 alpha, *ENL* eleven-nineteen leukemia, *eRNA* enhancer RNA, *EU* European Union, *FDA* US Food and Drug Administration, *GI* gastrointestinal, *HDAC* histone deacetylase, *HER2* human epidermal growth factor receptor 2, *HR* hormone receptor, *KRAB* Krüppel-associated box, *LSD1* lysine-specific demethylase 1, *MDS* myelodysplastic syndromes, *PD-1* programmed cell death protein 1, *PD-L1* programmed death-ligand 1, *Pol II* RNA polymerase II, *PTCL* peripheral T-cell lymphoma, *SE* super-enhancer, *siRNA* small interfering RNA

Recent efforts have sought to improve selectivity and therapeutic windows. More selective CDK7 and CDK9 inhibitors, as well as targeted protein degradation approaches, have been developed to reduce broad transcriptional suppression [216]. BET-directed proteolysis-targeting chimeras (PROTACs), including agents such as ARV-825 and MZ1, promote degradation of BET proteins rather than transient inhibition, leading to more sustained disruption of SE-associated transcription in preclinical models [217, 218]. However, these approaches remain constrained by on-target effects in normal tissues that also rely on SE-regulated transcriptional programs.

Combination strategies are being explored to improve efficacy and delay resistance. BET inhibition has been combined with CDK7 or CDK9 inhibition to target multiple layers of SE-dependent transcriptional control [165]. Histone deacetylase inhibitors may also alter enhancer acetylation states and increase dependence on BET-mediated transcription, providing a rationale for combination with BET inhibitors in selected contexts [219]. Nevertheless, such regimens require careful evaluation because broad inhibition of transcriptional regulators can produce dose-limiting toxicity. Biomarker-driven patient selection, based on SE dependency, transcriptional addiction or chromatin state, will be important for defining contexts in which these therapies are most likely to be effective [200].

### CRISPR-based epigenetic editing of SE activity

CRISPR-based epigenetic editing provides a complementary strategy for modulating SE activity without introducing double-strand DNA breaks or altering the underlying genomic sequence. Catalytically inactive Cas9 (dCas9) fused to chromatin-modifying domains can be directed to SE regions to either activate or repress enhancer function [203]. For example, dCas9-p300 or dCas9-CBP systems can deposit H3K27ac and enhance regulatory activity, whereas dCas9-KRAB or dCas9-MeCP2 can promote repressive chromatin states and reduce SE-driven transcription [220, 221]. These tools have been used to investigate enhancer function and could provide a useful experimental foundation for developing therapeutic approaches that modulate disease-associated SEs.

Additional platforms have expanded the range of epigenetic perturbations that can be applied to SEs. dCas9-LSD1 can reduce enhancer-associated histone H3 lysine 4 monomethylation (H3K4me1), whereas dCas9-DNMT3A can induce DNA methylation and potentially more durable transcriptional silencing [162, 204]. RNA-targeting CRISPR systems, including Cas13-based approaches, may also be used to reduce enhancer RNA levels and disrupt eRNA-associated chromatin interactions. Multiplexed CRISPR epigenome editing further enables simultaneous perturbation of multiple regulatory elements within a disease-associated transcriptional network [222].

Despite their conceptual advantages, CRISPR-based SE-targeting approaches remain largely preclinical [223]. Delivery efficiency, cell-type specificity, editing durability, immunogenicity, and off-target chromatin changes are persistent obstacles [224]. For now, these platforms are used mainly to define SE function mechanistically; advances in delivery and control of editor activity will determine whether they can support therapeutic applications [225].

### SE modulation in immunotherapy and tumor–immune interactions

SEs and SE-associated regulatory elements have been implicated in immune checkpoint expression, T cell differentiation and tumor immune evasion, suggesting potential opportunities for combining SE modulation with immunotherapy [226]. In chronically stimulated T cells, enhancer remodeling at exhaustion-related loci such as PDCD1 and HAVCR2 can help maintain inhibitory receptor expression [227]. Pharmacological or genetic perturbation of SE-associated transcriptional regulators may alter these exhaustion programs and improve effector function in selected experimental models [228].

In tumor cells, SE-associated regulatory elements can contribute to immune evasion by supporting expression of checkpoint molecules, including PD-L1, as well as genes involved in cytokine signaling and antigen presentation [211, 229]. Oncogenic pathways such as MYC, STAT3 and hypoxia-inducible signaling can converge on enhancer landscapes to regulate these immune-modulatory genes [230]. CRISPR-based epigenetic tools could, in principle, be used to repress checkpoint-associated regulatory elements or activate antigen-presentation programs, although the specificity and durability of these effects remain to be established [231].

Adoptive cell therapies may also benefit from improved control of SE-regulated transcriptional states [232]. Engineering strategies that limit exhaustion-associated regulatory programs or enhance persistence-associated transcriptional states could improve chimeric antigen receptor (CAR-T) cell function, particularly in solid tumors [233, 234]. However, the therapeutic manipulation of SEs in immune cells requires caution, as broad disruption of enhancer networks may impair normal immune activation, differentiation or tolerance. Future work should define which SE-associated immune programs are therapeutically actionable and which are required for normal immune homeostasis.

### Therapy-induced SE reprogramming, resistance, and retargeting opportunities

The same regulatory plasticity that creates therapeutic vulnerabilities can also enable escape after treatment. The following subsections therefore consider resistance as a consequence of treatment-imposed selection and adaptive enhancer rewiring, and then assess whether the resulting states create opportunities for biomarker-guided combination or sequential retargeting.

#### SE remodeling in endocrine and CDK4/6 inhibitor resistance

In luminal breast cancer, resistance to endocrine therapy often involves adaptive remodeling of estrogen receptor signaling and related regulatory landscapes [235]. ESR1-associated SEs, together with lineage-defining factors such as FOXA1, contribute to sustained ERα-dependent transcription under estrogen-responsive conditions. During prolonged exposure to endocrine therapies, including tamoxifen and aromatase inhibitors, resistant cells may acquire ESR1 mutations such as Y537S and D538G, which support ligand-independent ERα activity and maintain ERα-driven transcriptional programs despite estrogen deprivation [236, 237]. In parallel, enhancer remodeling at alternative growth factor signaling loci, including FGFR, IGF1R and ERBB3, may provide bypass proliferative signals and reduce dependence on canonical ERα signaling.

Resistance to CDK4/6 inhibitors can also involve transcriptional adaptation [238, 239]. In resistant cells, regulatory elements associated with cell-cycle control and E2F-dependent transcription may become reactivated, supporting expression of genes such as CCNE1 and CDK2 and restoring RB phosphorylation [240]. These changes can enable cell-cycle progression despite pharmacological CDK4/6 blockade [241]. Preclinical studies suggest that inhibition of SE-associated transcriptional regulators, including BET proteins or CDK7, may attenuate some resistance-associated transcriptional programs and restore sensitivity in selected models. However, the clinical relevance of these combinations requires further evaluation, particularly given the toxicity associated with broad transcriptional inhibition.

#### Adaptive SE rewiring during HER2-targeted therapy

HER2-positive breast cancer is treated with HER2-directed antibodies, tyrosine kinase inhibitors and antibody–drug conjugates, including trastuzumab deruxtecan (T-DXd) [242, 243]. Although these agents have improved clinical outcomes, acquired resistance remains common. Suppression of HER2 signaling can impose selective pressure on tumor cells and promote adaptive transcriptional states. In this context, enhancer or SE remodeling may support activation of alternative receptor tyrosine kinase and survival pathways, including EGFR, MET, AXL and downstream PI3K–AKT or MAPK signaling [244]. Such changes may reduce dependence on ERBB2-associated transcriptional programs and provide compensatory mechanisms for continued proliferation and survival.

The mechanisms of HER2-directed agents should be distinguished when considering their potential effects on enhancer regulation. Trastuzumab is an HER2-directed antibody without a cytotoxic payload, whereas trastuzumab emtansine (T-DM1) delivers the microtubule inhibitor DM1. By contrast, T-DXd is an antibody–drug conjugate that releases DXd, a membrane-permeable exatecan-derived DNA topoisomerase I (TOP1) inhibitor, following HER2-dependent internalization and linker cleavage [245]. DXd stabilizes TOP1–DNA cleavage complexes and induces replication-associated DNA damage, thereby linking T-DXd activity to DNA-damage and repair pathways [246].

Oncogenic SEs can experience transcription-coupled DNA breaks and engage TOP1- and RAD51-associated repair, providing a mechanistic basis for their potential sensitivity to perturbations of DNA topology and repair [247]. Nevertheless, direct evidence that T-DXd or free TOP1 inhibitors selectively disrupt an ERBB2-associated SE is currently lacking. In particular, it has not been established that these agents consistently reduce H3K27ac, BRD4 or MED1 occupancy, alter enhancer–promoter contacts or abolish the functional requirement for ERBB2 regulatory elements. Any effect of the DXd payload on HER2-associated enhancer activity should therefore be regarded as mechanistically plausible and context-dependent, rather than as an established component of T-DXd action.

#### SE-associated programs in chemotherapy resistance and cancer stemness

Beyond adaptive rewiring following targeted therapy, conventional DNA-targeting chemotherapy can also alter enhancer-associated transcription, although it does not exert a uniform effect on SEs [248–250]. Topoisomerase poisons, DNA-crosslinking agents and replication-stress-inducing drugs can acutely interfere with transcription, DNA topology and chromatin accessibility while activating DNA-damage and repair pathways [247, 251]. These immediate responses should be distinguished from the more persistent regulatory states that emerge through selection or adaptation during repeated treatment. Such states may retain pre-existing enhancer dependencies, acquire new enhancers or SEs, or alter enhancer–promoter communication to support survival [248–250]. Thus, reductions in global transcription following acute DNA damage do not necessarily predict the enhancer landscape of residual or drug-resistant tumor cells.

Evidence for treatment-associated enhancer reprogramming is strongest in selected experimental systems. In platinum-resistant ovarian cancer models, chemotherapy-induced distal enhancers and SEs maintain transcriptional programs that differ from those required in untreated cells, supporting a functional contribution to the resistant state [248]. Doxorubicin-resistant breast cancer cells similarly exhibit extensive changes in chromatin accessibility and three-dimensional genome organization, including altered enhancer–promoter interactions [249, 252]. SE-associated regulation may also intersect with cancer stem-like phenotypes, which can be enriched following chemotherapy and contribute to tumor persistence and relapse. In breast cancer stem-like cell models, HDAC7 contributes to H3K27ac enrichment at SEs and the expression of stemness- and plasticity-associated genes, including *MYC*, *CD44* and *SNAI2* (SLUG); direct depletion or HDAC1/HDAC3 inhibition attenuates these programs, at least partly through HDAC7 downregulation [253]. In ovarian cancer, BRD4-dependent regulation of an ALDH1A1-associated SE and its enhancer RNA sustains ALDH activity, whereas BET inhibition suppresses the outgrowth of cisplatin-treated cells in vitro and in vivo [163]. Conversely, BRD4 can also participate in SE-associated transcriptional repression, and disruption of a BRD4–LSD1–NuRD regulatory complex has been linked to broad drug resistance in breast cancer [254]. These findings caution against treating SE activity as uniformly pro-resistance or assuming that pharmacological disruption of SE-associated machinery will have predictable effects across contexts.

A central unresolved question is whether treatment-associated enhancer changes drive resistance or merely mark the cell populations that survive therapy. Addressing this distinction will require time-resolved profiling of H3K27ac, transcription-factor and cofactor occupancy, chromatin accessibility and genome topology before, during and after treatment, ideally in matched patient-derived samples. Locus-specific genetic or epigenetic perturbation should subsequently determine whether candidate enhancers, SEs or enhancer RNAs are required for drug efflux, DNA repair, anti-apoptotic signaling or maintenance of stem-like states. DNA-targeting chemotherapy should therefore be considered capable of reshaping enhancer-associated transcription in a drug-, dose-, time- and cell-state-dependent manner, rather than as uniformly activating or suppressing SEs.

## Challenges and future directions in SE research

Therapeutic studies highlight both the potential and the limitations of targeting SE-associated transcription. Context-dependent enhancer usage, incomplete target-gene assignment, model-specific dependencies, systemic toxicity, and treatment-induced regulatory plasticity continue to impede clinical translation. Addressing these barriers requires methodological standardization, longitudinal functional validation, and models that better capture cellular heterogeneity and therapeutic evolution. This section discusses these unresolved challenges and considers how single-cell and spatial profiling, computational integration, patient-derived models, and biomarker-guided strategies may improve the rigor and translational relevance of SE research.

### Definitional uncertainty, model limitations and data standardization

Several unresolved issues continue to limit the reproducibility and translational relevance of SE studies. A central challenge is the absence of a universally accepted definition of SEs [22]. Most studies rely on operational criteria based on H3K27ac enrichment, transcriptional cofactor occupancy and ranking-based algorithms such as ROSE [8]. However, these approaches define SEs relative to other enhancers within a given dataset rather than by fixed biological properties [6]. As a result, SE annotations are sensitive to peak calling, stitching distance, signal normalization and threshold selection. This variability complicates comparisons across cell types, disease states and independent studies.

Experimental models introduce additional limitations. Two-dimensional cell lines and patient-derived organoids provide tractable systems for perturbation studies, but they incompletely recapitulate immune, stromal, vascular and systemic regulatory inputs [52, 53]. Genetically engineered mouse models offer in vivo context, but species-specific differences in enhancer architecture and transcription factor binding can restrict direct extrapolation to human disease. Patient-derived xenograft models preserve aspects of human tumor biology but often lack intact immune and stromal compartments [61]. These limitations underscore the need to interpret SE function in light of model-specific constraints.

Data standardization remains a further barrier. Differences in ChIP-seq protocols, antibody performance, sequencing depth, sample purity and bioinformatic pipelines can produce divergent SE landscapes [35, 255]. Integrating scATAC-seq, scRNA-seq, spatial transcriptomics and chromatin interaction data introduces additional batch effects and platform-specific assumptions. Community-level reference datasets, quality-control standards and transparent computational workflows will be needed to improve reproducibility and facilitate cross-study validation [256].

### Single-cell, spatial and live-cell approaches

Single-cell technologies can resolve regulatory states obscured in heterogeneous tissues. scATAC-seq profiles cell-specific chromatin accessibility, whereas scRNA-seq measures transcriptional output; their integration can associate accessible regulatory elements with cell-state-specific gene expression [44, 257–260]. A practical workflow includes quality control and filtering based on transcription-start-site enrichment and doublet detection, followed by construction of peak-by-cell and gene-by-cell matrices, dimensionality reduction, clustering, cell-type annotation, pseudobulk peak calling and peak-to-gene inference. Batch correction should be evaluated to ensure that biological variation is retained across samples and platforms. However, chromatin accessibility alone does not define an H3K27ac-based SE. Candidate cell-state-specific SEs therefore require orthogonal profiling of enhancer-associated histone marks or cofactor occupancy, whereas demonstration of functional dependency ultimately requires locus-specific perturbation. In cancer, this framework may help identify rare cell states, resistant populations and lineage transitions that are obscured in bulk analyses.

Spatial transcriptomics adds anatomical context to these regulatory maps [45, 46]. Platforms such as 10 × Visium, multiplexed error-robust fluorescence in situ hybridization (MERFISH) and Slide-seq can localize gene expression programs within native tissue architecture, helping to distinguish tumor-cell-intrinsic programs from stromal, immune or hypoxia-associated signals. Although spatial transcriptomics does not directly measure SE activity, its integration with epigenomic and chromatin-accessibility data can support inference of spatially organized regulatory states and tumor–microenvironment interactions [24].

Complementary combinations of transcriptional profiling, chromatin-accessibility assays and structural imaging may further refine SE analysis. Super-resolution imaging and CRISPR–dCas9-based locus labeling can visualize locus positioning, enhancer–promoter proximity and their spatial relationship to transcriptional condensates [261, 262]. Live-cell imaging may additionally provide temporal information on regulatory dynamics during differentiation, stress responses and drug exposure [263, 264]. Nevertheless, spatial proximity or condensate association does not by itself establish a functional SE dependency and should be integrated with perturbation-based evidence.

### Artificial intelligence and computational integration

Artificial intelligence (AI), particularly machine learning, is increasingly being applied to SE research for regulatory network inference, drug prioritization and multi-omics integration [265]. Deep learning models can incorporate chromatin accessibility, histone modifications, transcription factor occupancy, chromatin interactions and gene expression to predict enhancer–promoter relationships and disease-associated regulatory circuits [266, 267]. Graph-based methods may be especially useful for modeling SE–promoter interactions within three-dimensional chromatin networks [268].

AI-assisted approaches may also support therapeutic discovery [269]. Computational models that integrate SE annotations with drug-response profiles can prioritize candidate vulnerabilities associated with transcriptional dependencies. In parallel, AI-guided approaches may support degrader and molecular-glue design, as well as the prioritization of rational combination regimens targeting SE-associated regulators, including BET proteins, CDK7, CDK9 and p300/CBP [270]. However, these predictions require rigorous experimental validation. Many models remain limited by training-data bias, incomplete annotation of non-coding regulatory elements and insufficient representation of patient-derived datasets [25].

Target discovery is also moving beyond candidate-based selection toward functional CRISPR screens, chemical–genetic profiling and multi-omic dependency mapping, whereas library generation increasingly includes focused epigenetic inhibitor sets, covalent-ligand collections and modular degrader libraries [19, 47, 216–218]. These approaches can broaden target and scaffold space, but biochemical target engagement and chromatin-level validation remain necessary before a hit can be interpreted as an SE-associated dependency.

For clinical translation, AI-based models should be evaluated against clinically annotated cohorts, longitudinal samples and treatment-response datasets [271, 272]. Predictive frameworks will be most useful when they integrate enhancer state with genomic alterations, tumor subtype, microenvironmental context and pharmacological sensitivity. Without such validation, computational predictions should be viewed as hypothesis-generating rather than clinically actionable [273].

### Translational priorities and next-generation therapeutic strategies

The clinical translation of SE-targeted therapies is limited by the fact that SEs also regulate essential transcriptional programs in normal tissues [166]. BET inhibitors can cause hematologic and gastrointestinal toxicities, whereas CDK7 and CDK9 inhibitors may affect bone marrow function and other rapidly renewing tissues [201]. These toxicities reflect the broad roles of transcriptional cofactors in normal physiology and underscore the need for selective dosing strategies, rational combinations and predictive biomarkers [167].

Delivery remains a central barrier for CRISPR-based or nucleic-acid-based SE modulation [205]. Lipid nanoparticles (LNPs) are among the most advanced non-viral delivery platforms and can package messenger RNA (mRNA), guide RNAs or ribonucleoprotein components [274, 275]. Their transient expression profile may reduce prolonged exposure and off-target editing. However, current LNP formulations often show preferential accumulation in the liver, spleen and bone marrow, limiting efficient delivery to many solid tumors [206]. Ligand modification may improve tumor targeting, but it also increases manufacturing complexity and may introduce additional safety considerations.

Virus-like particles (VLPs) provide an alternative platform for delivering genome or epigenome editing components as ribonucleoprotein complexes, potentially reducing risks associated with persistent nuclease expression or genomic integration [207, 276]. Engineered VLPs can be further optimized for cell-type-selective delivery, although improvements in in vivo stability, biodistribution, packaging efficiency and scalability are still needed [208, 277]. Overcoming these delivery and safety barriers will be essential before CRISPR-based SE modulation can be considered for routine therapeutic use.

These barriers define the priorities for next-generation intervention: disease- and cell-type-selective delivery, locus- or target-selective modulation, pharmacodynamic biomarkers that demonstrate engagement of the proposed regulatory dependency, and longitudinal sampling to detect compensatory enhancer rewiring. CRISPR-based epigenetic editing, targeted protein degradation and synthetic regulatory systems may improve precision, but remain largely preclinical and will require rigorous evaluation of durability, off-target chromatin effects, immunogenicity and manufacturability [278–280].

Future progress in SE research will depend on integrating mechanistic biology with clinically relevant models and standardized datasets [281]. Multi-omics approaches that combine SE maps with transcriptomics, proteomics, metabolomics, phosphoproteomics and genome-wide association data may help connect regulatory architecture with disease phenotypes [282]. Such integrative analyses could identify inherited or somatic variants that alter SE function and contribute to disease susceptibility, progression or treatment response [149].

SE-based biomarkers also require more rigorous development [283]. Tissue-based SE burden scores, circulating enhancer-derived RNAs, chromatin-state measurements from cell-free DNA and single-cell regulatory heterogeneity indices may provide complementary information, but their clinical value remains to be established [284]. Prospective validation will be necessary to determine whether these biomarkers can support patient stratification, treatment monitoring or early detection of therapeutic resistance [285].

## Conclusions and future perspectives

The super-enhancer (SE) framework has provided a useful means of linking dense enhancer occupancy to genes that support cell identity and context-dependent transcription. However, SEs do not constitute a mechanistically uniform class of enhancers. Individual SE loci can contain functionally heterogeneous elements, including classic enhancers, facilitators and partially redundant components. Furthermore, their annotation depends on relative epigenomic signal strength, computational ranking, stitching distance and threshold selection. Thus, SE rank or H3K27ac enrichment alone should not be interpreted as evidence for regulatory necessity. Mechanistic inference requires the integration of chromatin profiling with locus-specific perturbation and experimentally supported enhancer–promoter assignment.

Across physiology, SE-associated regulation has been implicated in lineage specification, trophoblast development and pancreatic β-cell function. In disease, altered SE or enhancer landscapes can accompany aberrant cellular states, but the strength of causal evidence varies considerably. Breast cancer illustrates this distinction: functional evidence supports selected ERα-associated and triple-negative breast cancer-specific regulatory circuits, whereas evidence for a functionally required ERBB2-associated SE remains comparatively limited. Similarly, SE remodeling has been reported in rheumatoid arthritis, whereas studies in neurological disease more often identify disease-associated regulatory elements or chromatin states without establishing SE dependence. Disease-associated SE maps should therefore be distinguished from regulatory dependencies that are required to maintain pathological phenotypes.

Therapeutic disruption of SE-associated transcription remains a promising but experimentally and clinically immature strategy. BET, CDK7 and CDK9 inhibitors can suppress transcriptionally demanding programs, but these agents act on widely shared regulatory machinery rather than selectively targeting individual SEs. Early-phase studies and selected later-stage trials demonstrate that these target classes have entered clinical evaluation, but do not in themselves establish that treatment responses are mediated by SE dependence. BET-targeted degraders may produce durable protein depletion in preclinical models, though their selectivity, pharmacokinetics and resistance mechanisms require further evaluation. CRISPR-based epigenetic editing offers greater locus specificity, but delivery, durability, off-target chromatin effects and cell-type restrictions remain substantial barriers. Clinical translation will therefore require mechanism-informed biomarkers and therapeutic windows that distinguish disease-associated dependencies from essential transcriptional programs in normal tissues.

Several research questions should now be prioritized. What quantitative, molecular or topological features distinguish a functionally required SE from a highly active but dispensable cluster of enhancers? How are constituent enhancers organized into hierarchical, cooperative or redundant relationships, and how do these relationships change with genetic perturbation or treatment? How stable are SE dependencies across cell states, anatomical regions, treatment histories and patient populations? How are enhancer networks assembled and maintained on extrachromosomal DNA, and do enhancer hijacking, ecDNA hubs or mitotic inheritance create context-specific transcriptional dependencies that contribute to tumor evolution or therapeutic adaptation (see Fig. 3)? Finally, can predictive biomarkers identify patients in whom the SE-associated transcriptional machinery can be perturbed without unacceptable effects on normal tissue homeostasis? Addressing these questions will require experimental designs that explicitly distinguish association, functional necessity and therapeutic action.

Progress will depend on standardized annotation criteria, transparent computational workflows and perturbation-based validation in physiologically relevant systems. Patient-derived organoids and xenografts can provide complementary information about tissue architecture, clonal heterogeneity and treatment response, although neither fully recapitulates human physiology. Integration of these models with single-cell, spatial and three-dimensional epigenomic approaches should help resolve SE activity across cellular, spatial and temporal scales. Computational and artificial intelligence-based models may aid regulatory network inference and candidate prioritization, but their outputs should remain hypothesis-generating until validated in independent, clinically annotated cohorts. Ultimately, the translational value of SE research will depend on moving beyond descriptive maps towards reproducible, mechanistically validated and context-specific regulatory dependencies.

## Data Availability

Not applicable.

## References

[1] Long HK, Prescott SL, Wysocka J. Ever-Changing Landscapes: Transcriptional Enhancers in Development and Evolution. Cell. 2016;167(5):1170–87. 10.1016/j.cell.2016.09.018. PMC5123704 27863239

[2] Neyret-Kahn H, Fontugne J, Meng XY, Groeneveld CS, Cabel L, Ye T, et al. Epigenomic mapping identifies an enhancer repertoire that regulates cell identity in bladder cancer through distinct transcription factor networks. Oncogene. 2023;42(19):1524–42. 10.1038/s41388-023-02662-1. PMC10162941 36944729

[3] Schoenfelder S, Fraser P. Long-range enhancer-promoter contacts in gene expression control. Nat Rev Genet. 2019;20(8):437–55. 10.1038/s41576-019-0128-0. 31086298

[4] Whyte WA, Orlando DA, Hnisz D, Abraham BJ, Lin CY, Kagey MH, et al. Master transcription factors and mediator establish super-enhancers at key cell identity genes. Cell. 2013;153(2):307–19. 10.1016/j.cell.2013.03.035. PMC3653129 23582322

[5] Hnisz D, Abraham BJ, Lee TI, Lau A, Saint-André V, Sigova AA, et al. Super-enhancers in the control of cell identity and disease. Cell. 2013;155(4):934–47. 10.1016/j.cell.2013.09.053. PMC3841062 24119843

[6] Blayney JW, Francis H, Rampasekova A, Camellato B, Mitchell L, Stolper R, et al. Super-enhancers include classical enhancers and facilitators to fully activate gene expression. Cell. 2023;186(26):5826-5839.e18. 10.1016/j.cell.2023.11.030. PMC10858684 38101409

[7] Pott S, Lieb JD. What are super-enhancers? Nat Genet. 2015;47(1):8–12. 10.1038/ng.3167. 25547603

[8] Kravchuk EV, Ashniev GA, Gladkova MG, Orlov AV, Vasileva AV, Boldyreva AV, et al. Experimental Validation and Prediction of Super-Enhancers: Advances and Challenges. Cells. 2023;12(8):1191. 10.3390/cells12081191. PMC10136858 37190100

[9] Lee BK, Jang YJ, Kim M, LeBlanc L, Rhee C, Lee J, et al. Super-enhancer-guided mapping of regulatory networks controlling mouse trophoblast stem cells. Nat Commun. 2019;10(1):4749. 10.1038/s41467-019-12720-6. PMC6802173 31628347

[10] Lai X, Li C, Tang X, Luo X, Wu F, Liang Y, et al. Super-enhancers in immune system regulation: mechanisms, pathological reprogramming, and therapeutic opportunities. Front Immunol. 2025;16:1652398. 10.3389/fimmu.2025.1652398. PMC12394480 40895527

[11] Hu M, Kim I, Morán I, Peng W, Sun O, Bonnefond A, et al. Multiple genetic variants at the SLC30A8 locus affect local super-enhancer activity and influence pancreatic β-cell survival and function. FASEB J. 2024;38(8):e23610. 10.1096/fj.202301700RR. PMC11108099 38661000

[12] Yang JH, Hansen AS. Enhancer selectivity in space and time: from enhancer-promoter interactions to promoter activation. Nat Rev Mol Cell Biol. 2024;25(7):574–91. 10.1038/s41580-024-00710-6. PMC11574175 38413840

[13] Sabari BR, Dall’Agnese A, Boija A, Klein IA, Coffey EL, Shrinivas K, et al. Coactivator condensation at super-enhancers links phase separation and gene control. Science. 2018;361(6400):eaar3958. 10.1126/science.aar3958. PMC6092193 29930091

[14] Napoli S, Munz N, Guidetti F, Bertoni F. Enhancer RNAs (eRNAs) in Cancer: The Jacks of All Trades. Cancers (Basel). 2022;14(8):1978. 10.3390/cancers14081978. PMC9030334 35454885

[15] Bradner JE, Hnisz D, Young RA. Transcriptional Addiction in Cancer. Cell. 2017;168(4):629–43. 10.1016/j.cell.2016.12.013. PMC5308559 28187285

[16] Huang H, Hu J, Maryam A, Huang Q, Zhang Y, Ramakrishnan S, et al. Defining super-enhancer landscape in triple-negative breast cancer by multiomic profiling. Nat Commun. 2021;12(1):2242. 10.1038/s41467-021-22445-0. PMC8046763 33854062

[17] Zheng ZZ, Xia L, Hu GS, Liu JY, Hu YH, Chen YJ, et al. Super-enhancer-controlled positive feedback loop BRD4/ERα-RET-ERα promotes ERα-positive breast cancer. Nucleic Acids Res. 2022;50(18):10230–48. 10.1093/nar/gkac778. PMC9561272 36124682

[18] Rojhannezhad M, Soltani BM, Vasei M, Ghorbanmehr N, Mowla SJ. Functional analysis of a putative HER2-associated expressed enhancer, Her2-Enhancer1, in breast cancer cells. Sci Rep. 2023;13(1):19516. 10.1038/s41598-023-46460-x. PMC10636096 37945744

[19] Lewis MW, King CM, Wisniewska K, Regner MJ, Coffey A, Kelly MR, et al. CRISPR Screening of Transcribed Super-Enhancers Identifies Drivers of Triple-Negative Breast Cancer Progression. Cancer Res. 2024;84(21):3684–700. 10.1158/0008-5472.Can-23-3995. PMC11534545 39186674

[20] Fulco CP, Munschauer M, Anyoha R, Munson G, Grossman SR, Perez EM, et al. Systematic mapping of functional enhancer-promoter connections with CRISPR interference. Science. 2016;354(6313):769–73. 10.1126/science.aag2445. PMC5438575 27708057

[21] Piha-Paul SA, Hann CL, French CA, Cousin S, Braña I, Cassier PA, et al. Phase 1 Study of Molibresib (GSK525762), a Bromodomain and Extra-Terminal Domain Protein Inhibitor, in NUT Carcinoma and Other Solid Tumors. JNCI Cancer Spectr. 2020;4(2):pkz093. 10.1093/jncics/pkz093. PMC7165800 32328561

[22] Ma H, Qu J, Pang Z, Luo J, Yan M, Xu W, et al. Super-enhancer omics in stem cell. Mol Cancer. 2024;23(1):153. 10.1186/s12943-024-02066-z. PMC11293198 39090713

[23] Regner MJ, Garcia-Recio S, Thennavan A, Wisniewska K, Mendez-Giraldez R, Felsheim B, et al. Defining the regulatory logic of breast cancer using single-cell epigenetic and transcriptome profiling. Cell Genom. 2025;5(2):100765. 10.1016/j.xgen.2025.100765. PMC11872555 39914387

[24] Deng Y, Bartosovic M, Ma S, Zhang D, Kukanja P, Xiao Y, et al. Spatial profiling of chromatin accessibility in mouse and human tissues. Nature. 2022;609(7926):375–83. 10.1038/s41586-022-05094-1. PMC9452302 35978191

[25] Hamamoto R, Takasawa K, Shinkai N, Machino H, Kouno N, Asada K, et al. Analysis of super-enhancer using machine learning and its application to medical biology. Brief Bioinform. 2023;24(3):bbad107. 10.1093/bib/bbad107. PMC10199775 36960780

[26] Lovén J, Hoke HA, Lin CY, Lau A, Orlando DA, Vakoc CR, et al. Selective inhibition of tumor oncogenes by disruption of super-enhancers. Cell. 2013;153(2):320–34. 10.1016/j.cell.2013.03.036. PMC3760967 23582323

[27] Zuin J, Roth G, Zhan Y, Cramard J, Redolfi J, Piskadlo E, et al. Nonlinear control of transcription through enhancer-promoter interactions. Nature. 2022;604(7906):571–7. 10.1038/s41586-022-04570-y. PMC9021019 35418676

[28] Lee R, Kang MK, Kim YJ, Yang B, Shim H, Kim S, et al. CTCF-mediated chromatin looping provides a topological framework for the formation of phase-separated transcriptional condensates. Nucleic Acids Res. 2022;50(1):207–26. 10.1093/nar/gkab1242. PMC8855298 34931241

[29] Zamudio AV, Dall’Agnese A, Henninger JE, Manteiga JC, Afeyan LK, Hannett NM, et al. Mediator Condensates Localize Signaling Factors to Key Cell Identity Genes. Mol Cell. 2019;76(5):753-766.e6. 10.1016/j.molcel.2019.08.016. PMC6898777 31563432

[30] Hay D, Hughes JR, Babbs C, Davies JOJ, Graham BJ, Hanssen L, et al. Genetic dissection of the α-globin super-enhancer in vivo. Nat Genet. 2016;48(8):895–903. 10.1038/ng.3605. PMC5058437 27376235

[31] Shin HY, Willi M, HyunYoo K, Zeng X, Wang C, Metser G, et al. Hierarchy within the mammary STAT5-driven Wap super-enhancer. Nat Genet. 2016;48(8):904–11. 10.1038/ng.3606. PMC4963296 27376239

[32] Fulco CP, Nasser J, Jones TR, Munson G, Bergman DT, Subramanian V, et al. Activity-by-contact model of enhancer-promoter regulation from thousands of CRISPR perturbations. Nat Genet. 2019;51(12):1664–9. 10.1038/s41588-019-0538-0. PMC6886585 31784727

[33] Yan S, Liu Z, Wang T, Sui Y, Wu X, Shen J, et al. Super-Enhancer Reprograming Driven by SOX9 and TCF7L2 Represents Transcription-Targeted Therapeutic Vulnerability for Treating Gallbladder Cancer. Adv Sci (Weinh). 2024;11(47):e2406448. 10.1002/advs.202406448. PMC11653766 39492805

[34] Wang Y, Zhao Y, Cao G, Jiang M, Yuan X, Li H, et al. STAT3 Facilitates Super Enhancer Formation to Promote Fibroblast-To-Myofibroblast Differentiation by the Analysis of ATAC-Seq, RNA-Seq and ChIP-Seq. J Cell Mol Med. 2025;29(11):e70639. 10.1111/jcmm.70639. PMC12135348 40464702

[35] Chu Q, Peng L, Brea L, Keo V, Lu X, Yu J, et al. Appropriate normalization is critical to improve reproducibility of tissue ChIP-seq. Serican J Med. 2025;2(3):23692. 10.17161/sjm.v2i3.23692. PMC12880172 41657940

[36] Fu Z, Jiang S, Sun Y, Zheng S, Zong L, Li P. Cut&Tag: a powerful epigenetic tool for chromatin profiling. Epigenetics. 2024;19(1):2293411. 10.1080/15592294.2023.2293411. PMC10730171 38105608

[37] Kaya-Okur HS, Wu SJ, Codomo CA, Pledger ES, Bryson TD, Henikoff JG, et al. CUT&Tag for efficient epigenomic profiling of small samples and single cells. Nat Commun. 2019;10(1):1930. 10.1038/s41467-019-09982-5. PMC6488672 31036827

[38] Cheng S, Miao B, Li T, Zhao G, Zhang B. Review and evaluate the bioinformatics analysis strategies of ATAC-seq and CUT&Tag data. Genomics Proteomics Bioinformatics. 2024;22(3):qzae054. 10.1093/gpbjnl/qzae054. PMC11464419 39255248

[39] Hansen TJ, Hodges E. ATAC-STARR-seq reveals transcription factor-bound activators and silencers within chromatin-accessible regions of the human genome. Genome Res. 2022;32(8):1529–41. 10.1101/gr.276766.122. PMC9435738 35858748

[40] Lafontaine DL, Yang L, Dekker J, Gibcus JH. Hi-C 3.0: improved protocol for genome-wide chromosome conformation capture. Curr Protoc. 2021;1(7):e198. 10.1002/cpz1.198. PMC8362010 34286910

[41] Di Giammartino DC, Polyzos A, Apostolou E. Assessing Specific Networks of Chromatin Interactions with HiChIP. Methods Mol Biol. 2022;2532:113–41. 10.1007/978-1-0716-2497-5_7. 35867248

[42] Mumbach MR, Rubin AJ, Flynn RA, Dai C, Khavari PA, Greenleaf WJ, et al. HiChIP: efficient and sensitive analysis of protein-directed genome architecture. Nat Methods. 2016;13(11):919–22. 10.1038/nmeth.3999. PMC5501173 27643841

[43] Ziffra RS, Kim CN, Ross JM, Wilfert A, Turner TN, Haeussler M, et al. Single-cell epigenomics reveals mechanisms of human cortical development. Nature. 2021;598(7879):205–13. 10.1038/s41586-021-03209-8. PMC8494642 34616060

[44] Dickmänken H, Wojno M, Mahieu L, Theunis K, Ekşi EC, Christiaens V, et al. Evaluating single-cell ATAC-seq atlasing technologies using sequence-to-function modeling. Nat Commun. 2026;17(1):1951. 10.1038/s41467-026-68742-4. PMC12929571 41571655

[45] Fan Y, Andrusivová Ž, Wu Y, Chai C, Larsson L, He M, et al. Expansion spatial transcriptomics. Nat Methods. 2023;20(8):1179–82. 10.1038/s41592-023-01911-1. PMC11078125 37349575

[46] Zhang D, Schroeder A, Yan H, Yang H, Hu J, Lee MYY, et al. Inferring super-resolution tissue architecture by integrating spatial transcriptomics with histology. Nat Biotechnol. 2024;42(9):1372–7. 10.1038/s41587-023-02019-9. PMC11260191 38168986

[47] Chardon FM, McDiarmid TA, Page NF, Daza RM, Martin BK, Domcke S, et al. Multiplex, single-cell CRISPRa screening for cell type specific regulatory elements. Nat Commun. 2024;15(1):8209. 10.1038/s41467-024-52490-4. PMC11411074 39294132

[48] Alshardan A, Alashban Y, Alahmadi M, Albshri A, Alanazi R, Alkhudhayr H, et al. Integrative single-cell multi-omics network analysis to elucidate epigenetic regulation in neurodevelopmental disorders. SLAS Technol. 2026;37:100395. 10.1016/j.slast.2026.100395. 41580083

[49] Li H, Cui L, Zhou M, Zou Q, Zhao Y, Lin H, et al. Single-cell multi-omics data reveal heterogeneity in liver tissue microenvironment induced by hypertension. Mol Ther Nucleic Acids. 2025;36(4):102696. 10.1016/j.omtn.2025.102696. PMC12450633 40989794

[50] Feng E, Zheng X, Zhu F, Xiang L, Liu C, Wang L, et al. Single-Cell Multi-Omics Reveal Gene Regulatory Mechanisms Underlying Cardiac Embryonic Development. Genes (Basel). 2026;17(4):414. 10.3390/genes17040414. PMC13115850 42074532

[51] Flores-García LC, Rubio K, Ibarra-Sierra E, Silva-Cázares MB, Palma-Flores C, López-Camarillo C. Epigenetic and Transcriptional Reprogramming in 3D Culture Models in Breast Cancer. Cancers (Basel). 2025;17(23):3830. 10.3390/cancers17233830. PMC12691298 41375031

[52] Zhang G, Liang Q, Wu Y, Wang Y. Insights on the differences between two- and three-dimensional culture systems in tumor models (Review). Int J Mol Med. 2025;56(5):185. 10.3892/ijmm.2025.5626. PMC12425351 40910266

[53] Drost J, Clevers H. Organoids in cancer research. Nat Rev Cancer. 2018;18(7):407–18. 10.1038/s41568-018-0007-6. 29692415

[54] Vlachogiannis G, Hedayat S, Vatsiou A, Jamin Y, Fernández-Mateos J, Khan K, et al. Patient-derived organoids model treatment response of metastatic gastrointestinal cancers. Science. 2018;359(6378):920–6. 10.1126/science.aao2774. PMC6112415 29472484

[55] Tuveson D, Clevers H. Cancer modeling meets human organoid technology. Science. 2019;364(6444):952–5. 10.1126/science.aaw6985. 31171691

[56] Younis M, Arjmandi T, Haque M, McClain K, Walter-Angelo T, Back F, et al. Establishment of Patient-Derived Organoids for Pediatric Cancer Research. Cancers (Basel). 2026;18(9):1465. 10.3390/cancers18091465. PMC13162899 42122261

[57] Sharpless NE, Depinho RA. The mighty mouse: genetically engineered mouse models in cancer drug development. Nat Rev Drug Discov. 2006;5(9):741–54. 10.1038/nrd2110. 16915232

[58] Rudin CM, Poirier JT, Byers LA, Dive C, Dowlati A, George J, et al. Molecular subtypes of small cell lung cancer: a synthesis of human and mouse model data. Nat Rev Cancer. 2019;19(5):289–97. 10.1038/s41568-019-0133-9. PMC6538259 30926931

[59] Lampreht Tratar U, Horvat S, Cemazar M. Transgenic Mouse Models in Cancer Research. Front Oncol. 2018;8:268. 10.3389/fonc.2018.00268. PMC6062593 30079312

[60] Zheng X, Carstens JL, Kim J, Scheible M, Kaye J, Sugimoto H, et al. Epithelial-to-mesenchymal transition is dispensable for metastasis but induces chemoresistance in pancreatic cancer. Nature. 2015;527(7579):525–30. 10.1038/nature16064. PMC4849281 26560028

[61] Abdolahi S, Ghazvinian Z, Muhammadnejad S, Saleh M, Asadzadeh Aghdaei H, Baghaei K. Patient-derived xenograft (PDX) models, applications and challenges in cancer research. J Transl Med. 2022;20(1):206. 10.1186/s12967-022-03405-8. PMC9088152 35538576

[62] Yoshida GJ. Applications of patient-derived tumor xenograft models and tumor organoids. J Hematol Oncol. 2020;13(1):4. 10.1186/s13045-019-0829-z. PMC6947974 31910904

[63] Kenkre R, Chapman OS, Chow EY, Luebeck J, Lo YY, Paul M, et al. Preservation and clonal behavior of extrachromosomal DNA in patient-derived xenograft models of childhood cancers. Genome Med. 2026;18(1):110. 10.1186/s13073-026-01676-0. PMC13403850 42210429

[64] Yadav V, Kilambi R, Rastogi A, Sharma D, Nagarajan P, Suroliya V, et al. Deciphering Cancer Evolution Through Genomic Profiling of Patient-Derived Xenograft Together with Matched Primary Gallbladder Cancer. Dig Dis Sci. 2026;71(4):1345–57. 10.1007/s10620-025-09471-4. 41207908

[65] Liu M, Yang X. Patient-derived xenograft models: Current status, challenges, and innovations in cancer research. Genes Dis. 2025;12(5):101520. 10.1016/j.gendis.2025.101520. PMC12179623 40548062

[66] Yang D, Beddows I, Tang H, Bai S, Cascio S, McGonigal SC, et al. A novel humanized immune stroma PDX cancer model for therapeutic studies. Cancer Immunol Immunother. 2026;75(4):117. 10.1007/s00262-026-04349-4. PMC13013731 41874706

[67] Jian C, Fu H, Wu W, Zhang N, Liu Z, Xia Z, et al. Harnessing PDX and PDX 2.0: the next-generation paradigm for precision oncology and translational breakthroughs. Mol Cancer. 2026;25(1):82. 10.1186/s12943-026-02594-w. PMC13015159 41699647

[68] Regner MJ, Wisniewska K, Garcia-Recio S, Thennavan A, Mendez-Giraldez R, Malladi VS, et al. A multi-omic single-cell landscape of human gynecologic malignancies. Mol Cell. 2021;81(23):4924-4941.e10. 10.1016/j.molcel.2021.10.013. PMC8642316 34739872

[69] Simon M, Stüve P, Schmidleithner L, Bittner S, Beumer N, Strieder N, et al. Single-cell chromatin accessibility and transposable element landscapes reveal shared features of tissue-residing immune cells. Immunity. 2024;57(8):1975-1993.e10. 10.1016/j.immuni.2024.06.015. 39047731

[70] Marderstein AR, De Zuani M, Moeller R, Bezney J, Padhi EM, Wong S, et al. Single-cell multi-omics map of human fetal blood in Down syndrome. Nature. 2024;634(8032):104–12. 10.1038/s41586-024-07946-4. PMC11446839 39322663

[71] Cui X, Yin Q, Gao Z, Li Z, Chen X, Lv H, et al. CREATE: cell-type-specific cis-regulatory element identification via discrete embedding. Nat Commun. 2025;16(1):4607. 10.1038/s41467-025-59780-5. PMC12085597 40382355

[72] Freund MM, deHaro-Arbona FJ, Baloul S, Torhorst A, Roussos C, Ruffridge AJ, et al. Pioneer-factor activity requires stable chromatin occupancy mediated by both sequence-specific binding and disordered protein domains. Sci Adv. 2026;12(25):eaef2244. 10.1126/sciadv.aef2244. PMC13281792 42319935

[73] Orlando KA, Grimm SA, Wade PA. Pioneering new enhancers by GATA3: role of facilitating transcription factors and chromatin remodeling. Nucleic Acids Res. 2025;53(11):gkaf473. 10.1093/nar/gkaf473. PMC12143596 40479714

[74] Oo JA, Warwick T, Pálfi K, Lam F, McNicoll F, Prieto-Garcia C, et al. Long non-coding RNAs direct the SWI/SNF complex to cell type-specific enhancers. Nat Commun. 2025;16(1):131. 10.1038/s41467-024-55539-6. PMC11695977 39747144

[75] Jones CA, Tansey WP, Weissmiller AM. Emerging Themes in Mechanisms of Tumorigenesis by SWI/SNF Subunit Mutation. Epigenet Insights. 2022;15:25168657221115656. 10.1177/25168657221115656. PMC9329810 35911061

[76] Chen Q, Yang B, Liu X, Zhang XD, Zhang L, Liu T. Histone acetyltransferases CBP/p300 in tumorigenesis and CBP/p300 inhibitors as promising novel anticancer agents. Theranostics. 2022;12(11):4935–48. 10.7150/thno.73223. PMC9274749 35836809

[77] Narita T, Ito S, Higashijima Y, Chu WK, Neumann K, Walter J, et al. Enhancers are activated by p300/CBP activity-dependent PIC assembly, RNAPII recruitment, and pause release. Mol Cell. 2021;81(10):2166-2182.e6. 10.1016/j.molcel.2021.03.008. 33765415

[78] Lu X, Peng L, Chu Q, Ye S, Liu M, Hussain M, et al. Targeting p300 and CBP abolishes HOXB13-loss-induced lipogenesis and tumor metastasis. JCI Insight. 2025;10(22):e195743. 10.1172/jci.insight.195743. PMC12643514 41277556

[79] Zheng ZZ, Chen L, Hu GS, Peng BL, Huang QX, Wang A, et al. Nuclear receptor ESRRA promotes ERα-positive breast cancer through dual action on super enhancers and promoters to regulate gene transcriptional programs. Sci Bull. 2025;70(22):3822–39. 10.1016/j.scib.2025.09.044. 41111053

[80] Tabe-Bordbar S, Song YJ, Lunt BJ, Alavi Z, Prasanth KV, Sinha S. Mechanistic analysis of enhancer sequences in the estrogen receptor transcriptional program. Commun Biol. 2024;7(1):719. 10.1038/s42003-024-06400-5. PMC11167054 38862711

[81] Jehanno C, Le Goff P, Habauzit D, Le Page Y, Lecomte S, Lecluze E, et al. Hypoxia and ERα transcriptional crosstalk is associated with endocrine resistance in breast cancer. Cancers (Basel). 2022;14(19):4934. 10.3390/cancers14194934. PMC9563995 36230857

[82] Hsieh TS, Cattoglio C, Slobodyanyuk E, Hansen AS, Darzacq X, Tjian R. Enhancer-promoter interactions and transcription are largely maintained upon acute loss of CTCF, cohesin, WAPL or YY1. Nat Genet. 2022;54(12):1919–32. 10.1038/s41588-022-01223-8. PMC9729117 36471071

[83] Dehingia B, Milewska M, Janowski M, Pękowska A. CTCF shapes chromatin structure and gene expression in health and disease. EMBO Rep. 2022;23(9):e55146. 10.15252/embr.202255146. PMC9442299 35993175

[84] Wei Z, Wang S, Xu Y, Wang W, Soares F, Ahmed M, et al. MYC reshapes CTCF-mediated chromatin architecture in prostate cancer. Nat Commun. 2023;14(1):1787. 10.1038/s41467-023-37544-3. PMC10063626 36997534

[85] Sollier E, Riedel A, Toprak UH, Wierzbinska JA, Weichenhan D, Schmid JP, et al. Enhancer hijacking discovery in acute myeloid leukemia by Pyjacker identifies MNX1 activation via deletion 7q. Blood Cancer Discov. 2025;6(4):343–63. 10.1158/2643-3230.BCD-24-0278. PMC12209774 40162972

[86] Lee D, Kang J, Kim A. TAD-dependent sub-TAD is required for enhancer-promoter interaction enabling the β-globin transcription. FASEB J. 2024;38(22):e70181. 10.1096/fj.202401526RR. PMC11698014 39545685

[87] Muto K, Yoshida D, Suzuki T, Yamamoto N, Inoue N, Murakami-Sekimata A, et al. Essential roles of CTCF binding sites at TAD boundaries in modulating chromatin interactions and transcriptional regulation at the Ifng locus. Front Immunol. 2025;16:1667851. 10.3389/fimmu.2025.1667851. PMC12511032 41080543

[88] Hnisz D, Weintraub AS, Day DS, Valton AL, Bak RO, Li CH, et al. Activation of proto-oncogenes by disruption of chromosome neighborhoods. Science. 2016;351(6280):1454–8. 10.1126/science.aad9024. PMC4884612 26940867

[89] Boeynaems S, Chong S, Gsponer J, Holt L, Milovanovic D, Mitrea DM, et al. Phase Separation in Biology and Disease; Current Perspectives and Open Questions. J Mol Biol. 2023;435(5):167971. 10.1016/j.jmb.2023.167971. PMC9970028 36690068

[90] Ahn JH, Davis ES, Daugird TA, Zhao S, Quiroga IY, Uryu H, et al. Phase separation drives aberrant chromatin looping and cancer development. Nature. 2021;595(7868):591–5. 10.1038/s41586-021-03662-5. PMC8647409 34163069

[91] Shen Y, Huang Z, Yang R, Chen Y, Wang Q, Gao L. Insights into Enhancer RNAs: Biogenesis and Emerging Role in Brain Diseases. Neuroscientist. 2023;29(2):166–76. 10.1177/10738584211046889. 34612730

[92] Wang Y, Zhang C, Wang Y, Liu X, Zhang Z. Enhancer RNA (eRNA) in human diseases. Int J Mol Sci. 2022;23(19):11582. 10.3390/ijms231911582. PMC9569849 36232885

[93] Patel AY, Zarrineh P, Tuffa NT, Sheth JH, Mohan S, Iqbal M, et al. Subtype-specific enhancer RNAs define transcriptional regulators and prognosis in breast cancers. Mol Oncol. 2026;20(7):1835–56. 10.1002/1878-0261.70214. PMC13352962 41769727

[94] Uttam V, Rana MK, Sethi G, Jain A. Long non-coding RNAs as regulators of immune signaling pathways in esophageal cancer. Biochem Pharmacol. 2026;250(Pt 1):117987. 10.1016/j.bcp.2026.117987. 42002235

[95] Ye W, Xiao J, Li J, Yang C, Li C, Xi Z, et al. Super-enhancer-associated lncRNA HDAC11-AS1 aggravates hepatocellular carcinoma progression by modulating HDAC11 and NUP210 expression via promoting super-enhancer activity. Cell Oncol (Dordr). 2026;49(4):96. 10.1007/s13402-026-01200-x. PMC13365274 42126774

[96] Yang Y, Wang TY, Fry J, Li Y, Meng Q, Guo Q, et al. Tumor-specific lncRNA IGF1R-AS1 trans-regulates chromatin interactions associated with oncogenic MYC signaling. Nat Commun. 2026;17(1):4171. 10.1038/s41467-026-70814-4. PMC13153188 41857039

[97] Bohrer C, Varon E, Peretz E, Reinitz G, Kinor N, Halle D, et al. CCAT1 lncRNA is chromatin-retained and post-transcriptionally spliced. Histochem Cell Biol. 2024;162(1–2):91–107. 10.1007/s00418-024-02294-w. PMC11227459 38763947

[98] Xiang JF, Yin QF, Chen T, Zhang Y, Zhang XO, Wu Z, et al. Human colorectal cancer-specific CCAT1-L lncRNA regulates long-range chromatin interactions at the MYC locus. Cell Res. 2014;24(5):513–31. 10.1038/cr.2014.35. PMC4011346 24662484

[99] Han Z, Li W. Enhancer RNA: What we know and what we can achieve. Cell Prolif. 2022;55(4):e13202. 10.1111/cpr.13202. PMC9055912 35170113

[100] Jakobsen ST, Jensen RAM, Madsen MS, Ravnsborg T, Vaagenso CS, Siersbæk MS, et al. MYC activity at enhancers drives prognostic transcriptional programs through an epigenetic switch. Nat Genet. 2024;56(4):663–74. 10.1038/s41588-024-01676-z. 38454021

[101] Xiao S, Huang Q, Ren H, Yang M. The mechanism and function of super enhancer RNA. Genesis. 2021;59(5–6):e23422. 10.1002/dvg.23422. 34028961

[102] Purshouse K, Pollard SM, Bickmore WA. Imaging extrachromosomal DNA (ecDNA) in cancer. Histochem Cell Biol. 2024;162(1–2):53–64. 10.1007/s00418-024-02280-2. PMC7616135 38625562

[103] Kraft K, Murphy SE, Jones MG, Shi Q, Bhargava-Shah A, Luong C, et al. Enhancer activation from transposable elements in extrachromosomal DNA. Nat Cell Biol. 2025;27(11):1914–24. 10.1038/s41556-025-01788-6. PMC12611757 41120733

[104] Hung KL, Yost KE, Xie L, Shi Q, Helmsauer K, Luebeck J, et al. ecDNA hubs drive cooperative intermolecular oncogene expression. Nature. 2021;600(7890):731–6. 10.1038/s41586-021-04116-8. PMC9126690 34819668

[105] Yan X, Mischel P, Chang H. Extrachromosomal DNA in cancer. Nat Rev Cancer. 2024;24(4):261–73. 10.1038/s41568-024-00669-8. PMC13643890 38409389

[106] Yi E, Gujar AD, Guthrie M, Kim H, Zhao D, Johnson KC, et al. Live-Cell Imaging Shows Uneven Segregation of Extrachromosomal DNA Elements and Transcriptionally Active Extrachromosomal DNA Hubs in Cancer. Cancer Discov. 2022;12(2):468–83. 10.1158/2159-8290.CD-21-1376. PMC8831456 34819316

[107] Wu S, Bafna V, Mischel PS. Extrachromosomal DNA (ecDNA) in cancer pathogenesis. Curr Opin Genet Dev. 2021;66:78–82. 10.1016/j.gde.2021.01.001. 33477016

[108] Dong Y, He Q, Chen X, Yang F, He L, Zheng Y. Extrachromosomal DNA (ecDNA) in cancer: mechanisms, functions, and clinical implications. Front Oncol. 2023;13:1194405. 10.3389/fonc.2023.1194405. PMC10338009 37448518

[109] Montuori G, Tu F, Qin D, Schmargon R, Rodriguez-Fos E, Helmsauer K, et al. Extrachromosomal DNA-Driven Oncogene Dosage Heterogeneity Promotes Rapid Adaptation to Therapy in MYCN-Amplified Cancers. Cancer Discov. 2025;15(10):2054–77. 10.1158/2159-8290.CD-24-1738. PMC12456741 40773595

[110] Yi E, Chamorro González R, Henssen AG, Verhaak RGW. Extrachromosomal DNA amplifications in cancer. Nat Rev Genet. 2022;23(12):760–71. 10.1038/s41576-022-00521-5. PMC9671848 35953594

[111] Tang J, Weiser NE, Wang G, Chowdhry S, Curtis EJ, Zhao Y, et al. Enhancing transcription-replication conflict targets ecDNA-positive cancers. Nature. 2024;635(8037):210–8. 10.1038/s41586-024-07802-5. PMC11540844 39506153

[112] Mohan DR, Borges KS, Finco I, LaPensee CR, Rege J, Solon AL, et al. β-Catenin-Driven Differentiation Is a Tissue-Specific Epigenetic Vulnerability in Adrenal Cancer. Cancer Res. 2023;83(13):2123–41. 10.1158/0008-5472.CAN-22-2712. PMC10330305 37129912

[113] Pulice JL, Meyerson M. Amplified dosage of the NKX2-1 lineage transcription factor controls its oncogenic role in lung adenocarcinoma. Mol Cell. 2025;85(7):1311-1329.e16. 10.1016/j.molcel.2025.03.001. PMC12262185 40139189

[114] Saha D, Animireddy S, Lee J, Thommen A, Murvin MM, Lu Y, et al. Enhancer switching in cell lineage priming is linked to eRNA, Brg1’s AT-hook, and SWI/SNF recruitment. Mol Cell. 2024;84(10):1855-1869.e5. 10.1016/j.molcel.2024.03.013. PMC11104297 38593804

[115] Kang R, Tan Z, Lang M, Jin L, Zhang Y, Zhang Y, et al. EnhFFL: A database of enhancer mediated feed-forward loops for human and mouse. Precis Clin Med. 2021;4(2):129–35. 10.1093/pcmedi/pbab006. PMC8982537 35694152

[116] Larsen SB, Cowley CJ, Sajjath SM, Barrows D, Yang Y, Carroll TS, et al. Establishment, maintenance, and recall of inflammatory memory. Cell Stem Cell. 2021;28(10):1758-1774.e8. 10.1016/j.stem.2021.07.001. PMC8500942 34320411

[117] Feng C, Song C, Jiang Y, Zhao J, Zhang J, Wang Y, et al. Landscape and significance of human super enhancer-driven core transcription regulatory circuitry. Mol Ther Nucleic Acids. 2023;32:385–401. 10.1016/j.omtn.2023.03.014. PMC10149290 37131406

[118] Riegel D, Romero-Fernández E, Simon M, Adenugba AR, Singer K, Mayr R, et al. Integrated single-cell profiling dissects cell-state-specific enhancer landscapes of human tumor-infiltrating CD8+ T cells. Mol Cell. 2023;83(4):622-636.e10. 10.1016/j.molcel.2022.12.029. 36657444

[119] Luo R, Yan J, Oh JW, Xi W, Shigaki D, Wong W, et al. Dynamic network-guided CRISPRi screen identifies CTCF-loop-constrained nonlinear enhancer gene regulatory activity during cell state transitions. Nat Genet. 2023;55(8):1336–46. 10.1038/s41588-023-01450-7. PMC11012226 37488417

[120] Hamagami N, Kapadia D, Barclay KM, Huang Y, Abduljawad N, Cheng Z, et al. State-specific enhancer landscapes govern microglial plasticity. Immunity. 2026;59(2):288-305.e11. 10.1016/j.immuni.2025.11.023. PMC13050238 41506265

[121] Lee H, Jaquish A, Fernandes S, Zhao B, Elitz A, Cook K, et al. Cell fate specification during respiratory development requires ARID1A-containing canonical BAF complex activity. Nat Commun. 2026;17(1):5489. 10.1038/s41467-026-71954-3. PMC13284238 42014719

[122] Ringel AR, Benetti N, Magg A, Groll F, Schöpflin R, Kühnlein M, et al. Temporal loss of En1 during limb development causes distinct phenotypes. Genes Dev. 2026;40(9–10):627–37. 10.1101/gad.353542.125. PMC13138323 41986232

[123] Hendrickson CL, Blitz IL, Hussein A, Paraiso KD, Cho JS, Klymkowsky MW, et al. Foxi2 and Sox3 are master transcription regulators that control ectoderm germ layer specification in Xenopus. PLoS Biol. 2025;23(11):e3003476. 10.1371/journal.pbio.3003476. PMC12599972 41187170

[124] Kessler S, Minoux M, Joshi O, Ben Zouari Y, Ducret S, Ross F, et al. A multiple super-enhancer region establishes inter-TAD interactions and controls Hoxa function in cranial neural crest. Nat Commun. 2023;14(1):3242. 10.1038/s41467-023-38953-0. PMC10241789 37277355

[125] Anderson KM, Anderson DM, McAnally JR, Shelton JM, Bassel-Duby R, Olson EN. Transcription of the non-coding RNA upperhand controls Hand2 expression and heart development. Nature. 2016;539(7629):433–6. 10.1038/nature20128. PMC5261552 27783597

[126] Hatton RD, Harrington LE, Luther RJ, Wakefield T, Janowski KM, Oliver JR, et al. A distal conserved sequence element controls Ifng gene expression by T cells and NK cells. Immunity. 2006;25(5):717–29. 10.1016/j.immuni.2006.09.007. 17070076

[127] Liu C, Nagashima H, Fernando N, Bass V, Gopalakrishnan J, Signorella S, et al. A CTCF-binding site in the Mdm1-Il22-Ifng locus shapes cytokine expression profiles and plays a critical role in early Th1 cell fate specification. Immunity. 2024;57(5):1005-1018.e7. 10.1016/j.immuni.2024.04.007. PMC11108081 38697116

[128] Wang Z, Dong W, Feng X, Xu X, An Y, Xiao H, et al. Multidimensional mapping of stimulation-responsive regulatory elements and candidate causal variants in T cell activation. Sci Adv. 2026;12(1):eady2539. 10.1126/sciadv.ady2539. PMC12757029 41477821

[129] Sato Y, Osada E, Manome Y. Non-canonical NFKB signaling endows suppressive function through FOXP3-dependent regulatory T cell program. Heliyon. 2023;9(12):e22911. 10.1016/j.heliyon.2023.e22911. PMC10730750 38125410

[130] Chen KY, Kibayashi T, Giguelay A, Hata M, Nakajima S, Mikami N, et al. Genome-wide CRISPR screen in human T cells reveals regulators of FOXP3. Nature. 2025;642(8066):191–200. 10.1038/s41586-025-08795-5. 40140585

[131] Suga K, Suto A, Tanaka S, Sugawara Y, Kageyama T, Ishikawa J, et al. TAp63, a methotrexate target in CD4+ T cells, suppresses Foxp3 expression and exacerbates autoimmune arthritis. JCI Insight. 2023;8(10):e164778. 10.1172/jci.insight.164778. PMC10322677 37212280

[132] Pan Y, Ichiyanagi K. Human Alu loci gain enhancer-type epigenetic modifications in monocytes during response to bacterial LPS. Epigenetics Chromatin. 2026;19(1):40. 10.1186/s13072-026-00686-x. PMC13584426 42432787

[133] Li D, Shi X, Yang Y, Deng Y, Chen D, Chen S, et al. Targeting BRD4 ameliorates experimental emphysema by disrupting super-enhancer in polarized alveolar macrophage. Respir Res. 2025;26(1):46. 10.1186/s12931-025-03120-0. PMC11786429 39891210

[134] Tanwar VS, Reddy MA, Dey S, Malek V, Lanting L, Chen Z, et al. Palmitic acid alters enhancers/super-enhancers near inflammatory and efferocytosis-associated genes in human monocytes. J Lipid Res. 2025;66(4):100774. 10.1016/j.jlr.2025.100774. PMC12002881 40068774

[135] Wang J, Xiao Q, Cai Y, Wang M, Chen C, Wang L, et al. ABCA1-Super Enhancer RNA Promotes Cholesterol Efflux, Reduces Macrophage-Mediated Inflammation and Atherosclerosis. JACC Basic Transl Sci. 2024;9(12):1388–405. 10.1016/j.jacbts.2024.08.005. PMC11733767 39822602

[136] Wang L, Wu J, Sramek M, Obayomi SMB, Gao P, Li Y, et al. Heterogeneous enhancer states orchestrate β cell responses to metabolic stress. Nat Commun. 2024;15(1):9361. 10.1038/s41467-024-53717-0. PMC11522703 39472434

[137] Davidson RK, Weaver SA, Casey N, Kanojia S, Hogarth E, Schneider Aguirre R, et al. The Chd4 subunit of the NuRD complex regulates Pdx1-controlled genes involved in β-cell function. J Mol Endocrinol. 2022;69(2):329–41. 10.1530/jme-22-0011. PMC9260723 35521759

[138] Aida Y, Kataoka K. CREB activates the MafA promoter through proximal E-boxes and a CCAAT motif in pancreatic β-cells. J Mol Endocrinol. 2024;73(3):e240023. 10.1530/jme-24-0023. 39189982

[139] Haberman N, Cheung R, Pizza G, Cvetesic N, Nagy D, Maude H, et al. Liver kinase B1 (LKB1) regulates the epigenetic landscape of mouse pancreatic beta cells. FASEB J. 2024;38(16):e23885. 10.1096/fj.202401078r. PMC11378476 39139039

[140] Yuan X, Lu H, Han M, Han K, Zhang Y, Liang P, et al. HCBP6-induced activation of brown adipose tissue and upregulated of BAT cytokines genes. J Therm Biol. 2022;109:103306. 10.1016/j.jtherbio.2022.103306. 36195403

[141] Xue P, Holloway N, Tran A, Lin F, Dinh J, Yang C, et al. A distal enhancer with ETV4 binding is critical for UCP1 expression and thermogenesis in brown fat. Genes Dev. 2025;39(13–14):808–25. 10.1101/gad.352748.125. PMC12211996 40324882

[142] Tian D, Zeng X, Gong Y, Zheng Y, Zhang J, Wu Z. HDAC1 inhibits beige adipocyte-mediated thermogenesis through histone crotonylation of Pgc1a/Ucp1. Cell Signal. 2023;111:110875. 10.1016/j.cellsig.2023.110875. 37640195

[143] Su D, Yang H, Li X, Jiang T, Zhan S, Zhong T, et al. A Functionally Conserved Enhancer is Critical for PGC1A Expression and Thermogenesis in Brown Fat. Adv Sci (Weinh). 2026;13(31):e18153. 10.1002/advs.202518153. PMC13588000 41869877

[144] Li S, Ji S, Yu Z, Liu H, Wu J. Super-enhancer-associated miR-1260b coordinates adipogenesis and metabolic programming in human adipose stem cells. Adipocyte. 2026;15(1):2638071. 10.1080/21623945.2026.2638071. PMC12990932 41823133

[145] Helmsauer K, Valieva ME, Ali S, Chamorro González R, Schöpflin R, Röefzaad C, et al. Enhancer hijacking determines extrachromosomal circular MYCN amplicon architecture in neuroblastoma. Nat Commun. 2020;11(1):5823. 10.1038/s41467-020-19452-y. PMC7669906 33199677

[146] Morton AR, Dogan-Artun N, Faber ZJ, MacLeod G, Bartels CF, Piazza MS, et al. Functional Enhancers Shape Extrachromosomal Oncogene Amplifications. Cell. 2019;179(6):1330-1341.e13. 10.1016/j.cell.2019.10.039. PMC7241652 31761532

[147] Sim N, Carter JM, Deka K, Tan BKT, Sim Y, Tan SM, et al. TWEAK/Fn14 signalling driven super-enhancer reprogramming promotes pro-metastatic metabolic rewiring in triple-negative breast cancer. Nat Commun. 2024;15(1):5638. 10.1038/s41467-024-50071-z. PMC11224303 38965263

[148] Jovanović B, Temko D, Stevens LE, Seehawer M, Fassl A, Murphy K, et al. Heterogeneity and transcriptional drivers of triple-negative breast cancer. Cell Rep. 2023;42(12):113564. 10.1016/j.celrep.2023.113564. PMC10842760 38100350

[149] Lu Z, Wang Z, Zhu W, Cai S, Sun X, Huang H. Multi-omics dissection of super-enhancer heterogeneity reveals subtype-specific transcriptional drivers in triple-negative breast cancer. Genomics. 2025;117(5):111097. 10.1016/j.ygeno.2025.111097. 40816630

[150] Hah N, Murakami S, Nagari A, Danko CG, Kraus WL. Enhancer transcripts mark active estrogen receptor binding sites. Genome Res. 2013;23(8):1210–23. 10.1101/gr.152306.112. PMC3730096 23636943

[151] Joosten SEP, Gregoricchio S, Stelloo S, Yapıcı E, Huang CF, Yavuz K, et al. Estrogen receptor 1 chromatin profiling in human breast tumors reveals high inter-patient heterogeneity with enrichment of risk SNPs and enhancer activity at most-conserved regions. Genome Res. 2024;34(4):539–55. 10.1101/gr.278680.123. PMC11146591 38719469

[152] Li W, Notani D, Ma Q, Tanasa B, Nunez E, Chen AY, et al. Functional roles of enhancer RNAs for oestrogen-dependent transcriptional activation. Nature. 2013;498(7455):516–20. 10.1038/nature12210. PMC3718886 23728302

[153] Verschoor N, Smid M, Jager A, Sleijfer S, Wilting SM, Martens JWM. Integrative whole-genome and transcriptome analysis of HER2-amplified metastatic breast cancer. Breast Cancer Res. 2023;25(1):145. 10.1186/s13058-023-01743-z. PMC10648326 37968696

[154] Lehmann BD, Bauer JA, Chen X, Sanders ME, Chakravarthy AB, Shyr Y, et al. Identification of human triple-negative breast cancer subtypes and preclinical models for selection of targeted therapies. J Clin Invest. 2011;121(7):2750–67. 10.1172/jci45014. PMC3127435 21633166

[155] Lehmann BD, Jovanović B, Chen X, Estrada MV, Johnson KN, Shyr Y, et al. Refinement of Triple-Negative Breast Cancer Molecular Subtypes: Implications for Neoadjuvant Chemotherapy Selection. PLoS ONE. 2016;11(6):e0157368. 10.1371/journal.pone.0157368. PMC4911051 27310713

[156] Wu Q, Tao X, Luo Y, Zheng S, Lin N, Xie X. A novel super-enhancer-related gene signature predicts prognosis and immune microenvironment for breast cancer. BMC Cancer. 2023;23(1):776. 10.1186/s12885-023-11241-2. PMC10439574 37596527

[157] Li Q, Lv X, Han C, Kong Y, Dai Z, Huo D, et al. Enhancer reprogramming promotes the activation of cancer-associated fibroblasts and breast cancer metastasis. Theranostics. 2022;12(17):7491–508. 10.7150/thno.75853. PMC9691365 36438487

[158] Wu Y, Yi Z, Li J, Wei Y, Feng R, Liu J, et al. FGFR blockade boosts T cell infiltration into triple-negative breast cancer by regulating cancer-associated fibroblasts. Theranostics. 2022;12(10):4564–80. 10.7150/thno.68972. PMC9254240 35832090

[159] Wang X, Li J, Song D, Wu Y, Liu J, Yi Z, et al. AEBP1 drives fibroblast-mediated T cell dysfunction in tumors. Nat Commun. 2025;16(1):8171. 10.1038/s41467-025-63659-w. PMC12402166 40890191

[160] Betancur PA, Abraham BJ, Yiu YY, Willingham SB, Khameneh F, Zarnegar M, et al. A CD47-associated super-enhancer links pro-inflammatory signalling to CD47 upregulation in breast cancer. Nat Commun. 2017;8:14802. 10.1038/ncomms14802. PMC5382276 28378740

[161] Stone JK, Kim JH, Vukadin L, Richard A, Giannini HK, Lim SS, et al. Hypoxia induces cancer cell-specific chromatin interactions and increases MALAT1 expression in breast cancer cells. J Biol Chem. 2019;294(29):11213–24. 10.1074/jbc.RA118.006889. PMC6643033 31167784

[162] Li M, Liu M, Han W, Wang Z, Han D, Patalano S, et al. LSD1 Inhibition Disrupts Super-Enhancer-Driven Oncogenic Transcriptional Programs in Castration-Resistant Prostate Cancer. Cancer Res. 2023;83(10):1684–98. 10.1158/0008-5472.CAN-22-2433. PMC10192194 36877164

[163] Yokoyama Y, Zhu H, Lee JH, Kossenkov AV, Wu SY, Wickramasinghe JM, et al. BET Inhibitors Suppress ALDH Activity by Targeting ALDH1A1 Super-Enhancer in Ovarian Cancer. Cancer Res. 2016;76(21):6320–30. 10.1158/0008-5472.Can-16-0854. PMC5117661 27803105

[164] Zhou J, Toh SHM, Tan TK, Balan K, Lim JQ, Tan TZ, et al. Super-enhancer-driven TOX2 mediates oncogenesis in Natural Killer/T Cell Lymphoma. Mol Cancer. 2023;22(1):69. 10.1186/s12943-023-01767-1. PMC10084643 37032358

[165] Thieme E, Bruss N, Sun D, Dominguez EC, Coleman D, Liu T, et al. CDK9 inhibition induces epigenetic reprogramming revealing strategies to circumvent resistance in lymphoma. Mol Cancer. 2023;22(1):64. 10.1186/s12943-023-01762-6. PMC10061728 36998071

[166] Zhang Y, Yin Q, Zhao B, Liu F, Li S, Cai J, et al. TQB3617, a bromodomain and extra-terminal inhibitor, in patients with relapsed or refractory lymphoma: A multicenter, phase 1 trial. Med. 2026;7(1):100893. 10.1016/j.medj.2025.100893. 41151587

[167] Yin M, Li X, Ye Z, Deng J, Zhang M, Li Z, et al. A Phase I Open-label Study of the Safety, Tolerability, and Pharmacokinetics of NHWD-870 HCl in Patients with Lymphoma and Other Advanced Solid Tumors. Recent Pat Anticancer Drug Discov. 2026. Online ahead of print. 10.2174/0115748928447241260617103928. 42393893

[168] Rampal RK, Grosicki S, Chraniuk D, Abruzzese E, Bose P, Gerds AT, et al. Pelabresib plus ruxolitinib for JAK inhibitor-naive myelofibrosis: a randomized phase 3 trial. Nat Med. 2025;31(5):1531–8. 10.1038/s41591-025-03572-3. PMC12092244 40065169

[169] Wu S, Jiang Y, Hong Y, Chu X, Zhang Z, Tao Y, et al. BRD4 PROTAC degrader ARV-825 inhibits T-cell acute lymphoblastic leukemia by targeting “Undruggable” Myc-pathway genes. Cancer Cell Int. 2021;21(1):230. 10.1186/s12935-021-01908-w. PMC8061034 33888130

[170] Higashijima Y, Kanki Y. Potential roles of super enhancers in inflammatory gene transcription. FEBS J. 2022;289(19):5762–75. 10.1111/febs.16089. 34173323

[171] Jadhav RR, Hu B, Ye Z, Sheth K, Li X, Greenleaf WJ, et al. Reduced chromatin accessibility to CD4 T cell super-enhancers encompassing susceptibility loci of rheumatoid arthritis. EBioMedicine. 2022;76:103825. 10.1016/j.ebiom.2022.103825. PMC8790491 35085847

[172] Yamagata K, Nakayamada S, Zhang T, Nguyen AP, Ohkubo N, Iwata S, et al. IL-6 production through repression of UBASH3A gene via epigenetic dysregulation of super-enhancer in CD4+ T cells in rheumatoid arthritis. Inflamm Regen. 2022;42(1):46. 10.1186/s41232-022-00231-9. PMC9632101 36324153

[173] Ai R, Laragione T, Hammaker D, Boyle DL, Wildberg A, Maeshima K, et al. Comprehensive epigenetic landscape of rheumatoid arthritis fibroblast-like synoviocytes. Nat Commun. 2018;9(1):1921. 10.1038/s41467-018-04310-9. PMC5953939 29765031

[174] Tsuchiya H, Ota M, Sumitomo S, Ishigaki K, Suzuki A, Sakata T, et al. Parsing multiomics landscape of activated synovial fibroblasts highlights drug targets linked to genetic risk of rheumatoid arthritis. Ann Rheum Dis. 2021;80(4):440–50. 10.1136/annrheumdis-2020-218189. 33139312

[175] Bae S, Kim K, Kang K, Kim H, Lee M, Oh B, et al. RANKL-responsive epigenetic mechanism reprograms macrophages into bone-resorbing osteoclasts. Cell Mol Immunol. 2023;20(1):94–109. 10.1038/s41423-022-00959-x. PMC9794822 36513810

[176] Guo G, Wang H, Tong X, Ye L, Shi X, Fang S, et al. Transcriptional Landscape of Enhancer RNAs in Peripheral Blood Mononuclear Cells from Patients with Systemic Lupus Erythematosus. J Inflamm Res. 2022;15:775–91. 10.2147/JIR.S331188. PMC8824297 35153501

[177] Ghodke-Puranik Y, Olferiev M, Crow MK. Systemic lupus erythematosus genetics: insights into pathogenesis and implications for therapy. Nat Rev Rheumatol. 2024;20(10):635–48. 10.1038/s41584-024-01152-2. 39232240

[178] Zhang Y, Day K, Absher DM. STAT3-mediated allelic imbalance of novel genetic variant Rs1047643 and B-cell-specific super-enhancer in association with systemic lupus erythematosus. Elife. 2022;11:e72837. 10.7554/elife.72837. PMC8884724 35188103

[179] Fan Y, Su M, Yu L, Chen S, Zheng K, Lin J, et al. Integrated transcriptomic and epigenomic profiling reveals conserved molecular subtypes across systemic autoimmune diseases. Ann Rheum Dis. 2026;85(10):2044–55. 10.1016/j.ard.2026.05.008. 42218077

[180] She LY, Li LY, Tang H, Yu Q, Gao FY, Zeng YQ, et al. OTUD1 positively regulates microglia neuroinflammation and promotes the pathogenesis of Alzheimer’s disease by deubiquitinating C/EBPβ. Acta Pharmacol Sin. 2025;46(10):2608–21. 10.1038/s41401-025-01566-y. PMC12460882 40335710

[181] Sun N, Victor MB, Park YP, Xiong X, Scannail AN, Leary N, et al. Human microglial state dynamics in Alzheimer’s disease progression. Cell. 2023;186(20):4386-4403.e29. 10.1016/j.cell.2023.08.037. PMC10644954 37774678

[182] Novikova G, Kapoor M, Tcw J, Abud EM, Efthymiou AG, Chen SX, et al. Integration of Alzheimer’s disease genetics and myeloid genomics identifies disease risk regulatory elements and genes. Nat Commun. 2021;12(1):1610. 10.1038/s41467-021-21823-y. PMC7955030 33712570

[183] Liu S, Cho M, Huang YN, Park T, Chaudhuri S, Rosewood TJ, et al. Multi-omics analysis for identifying cell-type-specific and bulk-level druggable targets in Alzheimer’s disease. J Transl Med. 2025;23(1):788. 10.1186/s12967-025-06739-1. PMC12257706 40653482

[184] Su C, Argenziano M, Lu S, Pippin JA, Pahl MC, Leonard ME, et al. 3D promoter architecture re-organization during iPSC-derived neuronal cell differentiation implicates target genes for neurodevelopmental disorders. Prog Neurobiol. 2021;201:102000. 10.1016/j.pneurobio.2021.102000. PMC8096691 33545232

[185] Kawamura A, Fujii K, Tamada K, Abe Y, Nitahara K, Iwasaki T, et al. Duplication of the autism-related gene Chd8 leads to behavioral hyperactivity and neurodevelopmental defects in mice. Nat Commun. 2025;16(1):4641. 10.1038/s41467-025-59853-5. PMC12106843 40419468

[186] Chen C, Guo S, Shi Y, Gu X, Xu Z, Chen Y, et al. Massively parallel characterization of non-coding de novo mutations in autism spectrum disorder. J Genet Genomics. 2025;52(10):1246–58. 10.1016/j.jgg.2025.07.008. 40738258

[187] Ibarra IL, Ratnu VS, Gordillo L, Hwang IY, Mariani L, Weinand K, et al. Comparative chromatin accessibility upon BDNF stimulation delineates neuronal regulatory elements. Mol Syst Biol. 2022;18(8):MSB202110473. 10.15252/msb.202110473. PMC9396287 35996956

[188] Fujinuma S, Nakatsumi H, Shimizu H, Sugiyama S, Harada A, Goya T, et al. FOXK1 promotes nonalcoholic fatty liver disease by mediating mTORC1-dependent inhibition of hepatic fatty acid oxidation. Cell Rep. 2023;42(5):112530. 10.1016/j.celrep.2023.112530. 37209098

[189] Sun L, Yue Z, Wang L. Research on the function of epigenetic regulation in the inflammation of non-alcoholic fatty liver disease. Life Med. 2024;3(4):lnae030. 10.1093/lifemedi/lnae030. PMC11749620 39872862

[190] Zailaie SA, Khoja BB, Siddiqui JJ, Mawardi MH, Heaphy E, Aljagthmi A, et al. Investigating the role of non-coding RNA in non-alcoholic fatty liver disease. Non-Coding RNA. 2024;10(1):10. 10.3390/ncrna10010010. PMC10891858 38392965

[191] Ren Q, Sun Q, Fu J. Dysfunction of autophagy in high-fat diet-induced non-alcoholic fatty liver disease. Autophagy. 2024;20(2):221–41. 10.1080/15548627.2023.2254191. PMC10813589 37700498

[192] Zhu Y, Zhang H, Jiang P, Xie C, Luo Y, Chen J. Transcriptional and epigenetic alterations in the progression of non-alcoholic fatty liver disease and biomarkers helping to diagnose non-alcoholic steatohepatitis. Biomedicines. 2023;11(3):970. 10.3390/biomedicines11030970. PMC10046227 36979950

[193] Miao XC, Li H, Li Q, Zhu L, Yu YQ, Ji JG, et al. Cell-type-specific cis-eQTLs in pancreatic cell types identify novel risk genes for type 2 diabetes. Brief Bioinform. 2025;26(5):bbaf531. 10.1093/bib/bbaf531. PMC12510404 41066696

[194] Sokolowski EK, Kursawe R, Selvam V, Bhuiyan RM, Thibodeau A, Zhao C, et al. Multi-omic human pancreatic islet endoplasmic reticulum and cytokine stress response mapping provides type 2 diabetes genetic insights. Cell Metab. 2024;36(11):2468-2488.e7. 10.1016/j.cmet.2024.09.006. PMC11798411 39383866

[195] Yang Z, Zhu S, Lyu CA, Fu H, Chen W, Qiu X, et al. BAF60C links nucleolar stress to β cell dysfunction in type 2 diabetes through controlling Reg3b mRNA decay. Dev Cell. 2026;61(5):976-994.e11. 10.1016/j.devcel.2026.02.006. 41806831

[196] Sui Y, Wang T, Mei Y, Zhu Y, Jiang W, Shen J, et al. Targeting Super-Enhancer-Driven Transcriptional Dependencies Suppresses Aberrant Hedgehog Pathway Activation and Overcomes Smoothened Inhibitor Resistance. Cancer Res. 2024;84(16):2690–706. 10.1158/0008-5472.CAN-23-3306. 38775809

[197] Chen Y, Ying Y, Ma W, Ma H, Shi L, Gao X, et al. Targeting the Epigenetic Reader ENL Inhibits Super-Enhancer-Driven Oncogenic Transcription and Synergizes with BET Inhibition to Suppress Tumor Progression. Cancer Res. 2024;84(8):1237–51. 10.1158/0008-5472.CAN-23-1836. 38241700

[198] Zengerle M, Chan KH, Ciulli A. Selective Small Molecule Induced Degradation of the BET Bromodomain Protein BRD4. ACS Chem Biol. 2015;10(8):1770–7. 10.1021/acschembio.5b00216. PMC4548256 26035625

[199] Coombes RC, Howell S, Lord SR, Kenny L, Mansi J, Mitri Z, et al. Dose escalation and expansion cohorts in patients with advanced breast cancer in a Phase I study of the CDK7-inhibitor samuraciclib. Nat Commun. 2023;14(1):4444. 10.1038/s41467-023-40061-y. PMC10366102 37488191

[200] Yuan J, Li X, Yu S. CDK7-dependent transcriptional addiction in bone and soft tissue sarcomas: present and future. Biochim Biophys Acta Rev Cancer. 2022;1877(2):188680. 10.1016/j.bbcan.2022.188680. 35051528

[201] Hanna GJ, Cote GM, Chugh R, Thomas JS, Malhotra J, Cutler RE, et al. Safety and Efficacy of a Selective Inhibitor of Cyclin-dependent Kinase 9 (KB-0742) in Patients with Recurrent or Metastatic Adenoid Cystic Carcinoma. Cancer Res Commun. 2025;5(5):767–73. 10.1158/2767-9764.crc-25-0015. PMC12056915 40277179

[202] Parveen R, Harihar D, Chatterji BP. Recent histone deacetylase inhibitors in cancer therapy. Cancer. 2023;129(21):3372–80. 10.1002/cncr.34974. 37560925

[203] Gemberling MP, Siklenka K, Rodriguez E, Tonn-Eisinger KR, Barrera A, Liu F, et al. Transgenic mice for in vivo epigenome editing with CRISPR-based systems. Nat Methods. 2021;18(8):965–74. 10.1038/s41592-021-01207-2. PMC8349887 34341582

[204] Nuñez JK, Chen J, Pommier GC, Cogan JZ, Replogle JM, Adriaens C, et al. Genome-wide programmable transcriptional memory by CRISPR-based epigenome editing. Cell. 2021;184(9):2503-2519.e17. 10.1016/j.cell.2021.03.025. PMC8376083 33838111

[205] Simonsen JB. Lipid nanoparticle-based strategies for extrahepatic delivery of nucleic acid therapies - challenges and opportunities. J Control Release. 2024;370:763–72. 10.1016/j.jconrel.2024.04.022. 38621638

[206] Chen K, Han H, Zhao S, Xu B, Yin B, Lawanprasert A, et al. Lung and liver editing by lipid nanoparticle delivery of a stable CRISPR-Cas9 ribonucleoprotein. Nat Biotechnol. 2025;43(9):1445–57. 10.1038/s41587-024-02437-3. PMC12000389 39415058

[207] Ju S, Lee JH, Yang J, Jeong TY, Choi CG, Lee Y, et al. A versatile VLP-mediated CRISPR-RNP platform for precise genome editing and durable epigenome silencing in cancer. Mol Ther Nucleic Acids. 2026;37(3):102978. 10.1016/j.omtn.2026.102978. PMC13316571 42381707

[208] Lu C, Li Y, Cummings JR, Banskota S. Delivery of genome editors with engineered virus-like particles. Methods Enzymol. 2025;712:475–516. 10.1016/bs.mie.2025.01.007. 40121085

[209] Richter GHS, Hensel T, Schmidt O, Saratov V, von Heyking K, Becker-Dettling F, et al. Combined Inhibition of Epigenetic Readers and Transcription Initiation Targets the EWS-ETS Transcriptional Program in Ewing Sarcoma. Cancers (Basel). 2020;12(2):304. 10.3390/cancers12020304. PMC7072515 32012890

[210] Jiang YY, Jiang Y, Li CQ, Zhang Y, Dakle P, Kaur H, et al. TP63, SOX2, and KLF5 Establish a Core Regulatory Circuitry That Controls Epigenetic and Transcription Patterns in Esophageal Squamous Cell Carcinoma Cell Lines. Gastroenterology. 2020;159(4):1311-1327.e19. 10.1053/j.gastro.2020.06.050. 32619460

[211] Shi C, Chen L, Pi H, Cui H, Fan C, Tan F, et al. Identifying a locus in super-enhancer and its resident NFE2L1/MAFG as transcriptional factors that drive PD-L1 expression and immune evasion. Oncogenesis. 2023;12(1):56. 10.1038/s41389-023-00500-3. PMC10662283 37985752

[212] Alečković M, Li Z, Zhou N, Qiu X, Lulseged B, Foidart P, et al. Combination Therapies to Improve the Efficacy of Immunotherapy in Triple-negative Breast Cancer. Mol Cancer Ther. 2023;22(11):1304–18. 10.1158/1535-7163.MCT-23-0303. PMC10618734 37676980

[213] Ny L, Jespersen H, Karlsson J, Alsén S, Filges S, All-Eriksson C, et al. The PEMDAC phase 2 study of pembrolizumab and entinostat in patients with metastatic uveal melanoma. Nat Commun. 2021;12(1):5155. 10.1038/s41467-021-25332-w. PMC8397717 34453044

[214] Bewersdorf JP, Shallis RM, Sharon E, Park S, Ramaswamy R, Roe CE, et al. A multicenter phase Ib trial of the histone deacetylase inhibitor entinostat in combination with pembrolizumab in patients with myelodysplastic syndromes/neoplasms or acute myeloid leukemia refractory to hypomethylating agents. Ann Hematol. 2024;103(1):105–16. 10.1007/s00277-023-05552-4. PMC11838822 38036712

[215] Hu C, Shen L, Zou F, Wu Y, Wang B, Wang A, et al. Predicting and overcoming resistance to CDK9 inhibitors for cancer therapy. Acta Pharm Sin B. 2023;13(9):3694–707. 10.1016/j.apsb.2023.05.026. PMC10502288 37719386

[216] Li Y, Bao K, Sun J, Ge R, Zhang Q, Zhang B, et al. Design of PROTACs utilizing the E3 ligase GID4 for targeted protein degradation. Nat Struct Mol Biol. 2025;32(9):1825–37. 10.1038/s41594-025-01537-1. 40295770

[217] Wang X, Song J, Zhao Y, Li W, Li J, Xu X, et al. Overcoming breast cancer resistance through targeted protein degradation and next generation chimeras. Pharmacol Res. 2026;229:108239. 10.1016/j.phrs.2026.108239. 42107508

[218] He S, Gao F, Ma J, Ma H, Dong G, Sheng C. Aptamer-PROTAC Conjugates (APCs) for Tumor-Specific Targeting in Breast Cancer. Angew Chem Int Ed Engl. 2021;60(43):23299–305. 10.1002/anie.202107347. 34240523

[219] Doskey LC, Scholtz CR, Vail NR, Khanal S, Lee AL, Kandanur SGS, et al. Efficacy and Toxicity Analysis of Selective BET Bromodomain Inhibitors in Models of Inflammatory Liver Disease. J Med Chem. 2025;68(8):8091–105. 10.1021/acs.jmedchem.4c02555. PMC12731298 40227166

[220] Saberian M, Roosta A, Afrisham R. CRISPR-dCas9 epigenetic reprogramming in cancer: platforms, immuno-modulation and delivery challenges. Gene. 2026;1005:150246. 10.1016/j.gene.2026.150246. 42229577

[221] Cai R, Lv R, Shi X, Yang G, Jin J. CRISPR/dCas9 Tools: Epigenetic Mechanism and Application in Gene Transcriptional Regulation. Int J Mol Sci. 2023;24(19):14865. 10.3390/ijms241914865. PMC10573330 37834313

[222] Eskandani NA, Mirzaee D, Ramezani Farani M, Hatami A, Hatami M, Ghasemzaei M, et al. Light-controlled CRISPR-dCas9 epigenome editing: advanced drug-delivery strategies and oncology applications. Adv Drug Deliv Rev. 2026;236:115921. 10.1016/j.addr.2026.115921. 42342132

[223] Guo Z, Zhu AT, Fang RH, Zhang L. Viral and nonviral nanocarriers for in vivo CRISPR-based gene editing. Nano Res. 2024;17(10):8904–25. 10.1007/s12274-024-6748-5. PMC12341846 40810089

[224] Kazemian P, Yu SY, Thomson SB, Birkenshaw A, Leavitt BR, Ross CJD. Lipid-Nanoparticle-Based Delivery of CRISPR/Cas9 Genome-Editing Components. Mol Pharm. 2022;19(6):1669–86. 10.1021/acs.molpharmaceut.1c00916. PMC9176214 35594500

[225] Wang S, Kong H, Zhuo C, Liu L, Lv S, Cheng D, et al. Functionalized extracellular nanovesicles as advanced CRISPR delivery systems. Biomater Sci. 2024;12(14):3480–99. 10.1039/d4bm00054d. 38808607

[226] Xie F, Zhou X, Li H, Su P, Liu S, Li R, et al. USP8 promotes cancer progression and extracellular vesicle-mediated CD8+ T cell exhaustion by deubiquitinating the TGF-β receptor TβRII. EMBO J. 2022;41(16):e108791. 10.15252/embj.2021108791. PMC9379553 35811497

[227] Jerin C, Seo W, Nishikawa H. Genetic disruption of Pdcd-1 upstream enhancer boosts T cell function and antitumor responses. Immunol Lett. 2026;280:107171. 10.1016/j.imlet.2026.107171. 41905426

[228] Hu W, Zhu S, Chen Q, Badovinac VP, Peng W, Xue HH. Exhausted CD8+ T cell fate is programmed by dynamic CTCF-mediated enhancer activation and invariant CTCF-imposed barriers. Nat Immunol. 2026;27(8):1693–707. 10.1038/s41590-026-02585-5. PMC13674790 42409949

[229] Fan Z, Wu C, Chen M, Jiang Y, Wu Y, Mao R, et al. The generation of PD-L1 and PD-L2 in cancer cells: From nuclear chromatin reorganization to extracellular presentation. Acta Pharm Sin B. 2022;12(3):1041–53. 10.1016/j.apsb.2021.09.010. PMC9069407 35530130

[230] Jeong H, Koh J, Kim S, Yim J, Song SG, Kim H, et al. Cell-intrinsic PD-L1 signaling drives immunosuppression by myeloid-derived suppressor cells through IL-6/Jak/Stat3 in PD-L1-high lung cancer. J Immunother Cancer. 2025;13(3):e010612. 10.1136/jitc-2024-010612. PMC11887297 40050048

[231] Wei X, Liu J, Cheng J, Cai W, Xie W, Wang K, et al. Super-enhancer-driven ZFP36L1 promotes PD-L1 expression in infiltrative gastric cancer. Elife. 2024;13:e96445. 10.7554/eLife.96445. PMC11458174 39373630

[232] Datlinger P, Pankevich EV, Arnold CD, Pranckevicius N, Lin J, Romanovskaia D, et al. Systematic discovery of CRISPR-boosted CAR T cell immunotherapies. Nature. 2025;646(8086):963–72. 10.1038/s41586-025-09507-9. PMC12545207 40993398

[233] Fourfouris T, Lee KJ, Hurwitz S, Ahdoot A, Lee A, Zarrabi M, et al. Wnt signaling as a regulator of memory T cells: implications for CAR-T cell therapy. Front Immunol. 2026;17:1843548. 10.3389/fimmu.2026.1843548. PMC13212174 42212136

[234] Rakhshandehroo T, Mantri SR, Moravej H, Louis BBV, Salehi Farid A, Munaretto L, et al. A CAR enhancer increases the activity and persistence of CAR T cells. Nat Biotechnol. 2025;43(6):948–59. 10.1038/s41587-024-02339-4. PMC11779983 39079964

[235] Yu S, Wang Y, Gong X, Fan Z, Wang Z, Liang Z, et al. LncRNA AGPG Confers Endocrine Resistance in Breast Cancer by Promoting E2F1 Activity. Cancer Res. 2023;83(19):3220–36. 10.1158/0008-5472.CAN-23-0015. 37463119

[236] Venetis K, Pepe F, Pescia C, Cursano G, Criscitiello C, Frascarelli C, et al. ESR1 mutations in HR+/HER2-metastatic breast cancer: Enhancing the accuracy of ctDNA testing. Cancer Treat Rev. 2023;121:102642. 10.1016/j.ctrv.2023.102642. 37864956

[237] Sivasailam A, Kumar KS, Jayaprasad AG, Jancy SV, Harikumar A, Unnikrishnan PS, et al. Estrogen receptor alpha dynamics and plasticity during endocrine resistance. Biol Direct. 2025;20(1):70. 10.1186/s13062-025-00653-8. PMC12164206 40514718

[238] Cui Y, Li Y, Xu Y, Liu X, Kang X, Zhu J, et al. SLC7A11 protects luminal A breast cancer cells against ferroptosis induced by CDK4/6 inhibitors. Redox Biol. 2024;76:103304. 10.1016/j.redox.2024.103304. PMC11378944 39153252

[239] Zhang Y, Zhou S, Kai Y, Zhang YQ, Peng C, Li Z, et al. O-GlcNAcylation of MITF regulates its activity and CDK4/6 inhibitor resistance in breast cancer. Nat Commun. 2024;15(1):5597. 10.1038/s41467-024-49875-w. PMC11222436 38961064

[240] Chen MK, Luo L, Massoumi N, Keyomarsi K. Targeting CDK2 and other novel cell cycle targets for breast cancer therapy. Expert Opin Ther Targets. 2025;29(7):435–56. 10.1080/14728222.2025.2537412. PMC12535411 40692275

[241] Yang Y, Liao J, Pan Z, Meng J, Zhang L, Shi W, et al. Dual Inhibition of CDK4/6 and CDK7 Suppresses Triple-Negative Breast Cancer Progression via Epigenetic Modulation of SREBP1-Regulated Cholesterol Metabolism. Adv Sci (Weinh). 2025;12(5):e2413103. 10.1002/advs.202413103. PMC11791979 39656925

[242] Nur Husna SM, Wong KK. Margetuximab and trastuzumab deruxtecan: New generation of anti-HER2 immunotherapeutic agents for breast cancer. Mol Immunol. 2022;152:45–54. 10.1016/j.molimm.2022.10.005. 36272249

[243] Hu D, Lyu X, Li Z, Ekambaram P, Dongre A, Freeman T, et al. p95HER2, a truncated form of the HER2 oncoprotein, drives an immunosuppressive program in HER2+ breast cancer that limits trastuzumab deruxtecan efficacy. Nat Cancer. 2025;6(7):1202–22. 10.1038/s43018-025-00969-4. PMC13218304 40579589

[244] Marquez-Palencia M, Reza Herrera L, Parida PK, Ghosh S, Kim K, Das NM, et al. AXL/WRNIP1 Mediates Replication Stress Response and Promotes Therapy Resistance and Metachronous Metastasis in HER2+ Breast Cancer. Cancer Res. 2024;84(5):675–87. 10.1158/0008-5472.CAN-23-1459. PMC11221606 38190717

[245] Ogitani Y, Aida T, Hagihara K, Yamaguchi J, Ishii C, Harada N, et al. DS-8201a, A Novel HER2-Targeting ADC with a Novel DNA Topoisomerase I Inhibitor, Demonstrates a Promising Antitumor Efficacy with Differentiation from T-DM1. Clin Cancer Res. 2016;22(20):5097–108. 10.1158/1078-0432.Ccr-15-2822. 27026201

[246] Lee J, Kida K, Koh J, Liu H, Manyam GC, Gi YJ, et al. The DNA repair pathway as a therapeutic target to synergize with trastuzumab deruxtecan in HER2-targeted antibody-drug conjugate-resistant HER2-overexpressing breast cancer. J Exp Clin Cancer Res. 2024;43(1):236. 10.1186/s13046-024-03143-3. PMC11337831 39164784

[247] Hazan I, Monin J, Bouwman BAM, Crosetto N, Aqeilan RI. Activation of oncogenic super-enhancers is coupled with DNA repair by RAD51. Cell Rep. 2019;29(3):560-572.e4. 10.1016/j.celrep.2019.09.001. PMC6899447 31618627

[248] Shang S, Yang J, Jazaeri AA, Duval AJ, Tufan T, Lopes Fischer N, et al. Chemotherapy-induced distal enhancers drive transcriptional programs to maintain the chemoresistant state in ovarian cancer. Cancer Res. 2019;79(18):4599–611. 10.1158/0008-5472.Can-19-0215. PMC6744966 31358529

[249] Wang X, Yan J, Ye Z, Zhang Z, Wang S, Hao S, et al. Reorganization of 3D chromatin architecture in doxorubicin-resistant breast cancer cells. Front Cell Dev Biol. 2022;10:974750. 10.3389/fcell.2022.974750. PMC9393755 36003143

[250] Wei D, Yuan L, Xu X, Wu C, Huang Y, Zhang L, et al. Exploring epigenetic dynamics unveils a super-enhancer-mediated NDRG1-β-catenin axis in modulating gemcitabine resistance in pancreatic cancer. Cancer Lett. 2024;605:217284. 10.1016/j.canlet.2024.217284. 39366545

[251] Canela A, Maman Y, Huang SN, Wutz G, Tang W, Zagnoli-Vieira G, et al. Topoisomerase II-induced chromosome breakage and translocation is determined by chromosome architecture and transcriptional activity. Mol Cell. 2019;75(2):252-266.e8. 10.1016/j.molcel.2019.04.030. PMC8170508 31202577

[252] Wang X, Yan J, Shen B, Wei G. Integrated Chromatin Accessibility and Transcriptome Landscapes of Doxorubicin-Resistant Breast Cancer Cells. Front Cell Dev Biol. 2021;9:708066. 10.3389/fcell.2021.708066. PMC8363264 34395436

[253] Caslini C, Hong S, Ban YJ, Chen XS, Ince TA. HDAC7 regulates histone 3 lysine 27 acetylation and transcriptional activity at super-enhancer-associated genes in breast cancer stem cells. Oncogene. 2019;38(39):6599–614. 10.1038/s41388-019-0897-0. 31375747

[254] Liu B, Liu X, Han L, Chen X, Wu X, Wu J, et al. BRD4-directed super-enhancer organization of transcription repression programs links to chemotherapeutic efficacy in breast cancer. Proc Natl Acad Sci U S A. 2022;119(6):e2109133119. 10.1073/pnas.2109133119. PMC8832982 35105803

[255] Cao Y, Patel L, Alcoser L, Mendenhall E, Benner C, Heinz S, et al. Automated chromatin profiling with spa-ChIP-seq uncovers the impacts of condition variations. Genome Res. 2026;36(1):129–41. 10.1101/gr.281320.125. PMC12758389 41386983

[256] Xiao K, Zhang R, Tu J. Evaluating Reproducibility and Best Practices for Replicate Design in G-Quadruplex ChIP-Seq Studies. Int J Mol Sci. 2025;26(19):9769. 10.3390/ijms26199769. PMC12524710 41097033

[257] Li Y, Ullah S, Xian Y, Sun Y, Zheng Z, Ma X, et al. DbscATAC: a resource of single-cell super-enhancers/enhancers and gene markers derived from scATAC-seq data. Bioinformatics. 2025;41(7):btaf364. 10.1093/bioinformatics/btaf364. PMC12237509 40581357

[258] Tian C, Dong Y, Sun X, Xu L, Ma X, Tao Y, et al. Integrative scATAC-seq and mtDNA mutation analysis reveals disease-driven regulatory aberrations in AML. Sci Bull (Beijing). 2025;70(19):3215–32. 10.1016/j.scib.2025.07.009. 40781032

[259] Granja JM, Corces MR, Pierce SE, Bagdatli ST, Choudhry H, Chang HY, et al. ArchR is a scalable software package for integrative single-cell chromatin accessibility analysis. Nat Genet. 2021;53(3):403–11. 10.1038/s41588-021-00790-6. PMC8012210 33633365

[260] Stuart T, Srivastava A, Madad S, Lareau CA, Satija R. Single-cell chromatin state analysis with Signac. Nat Methods. 2021;18(11):1333–41. 10.1038/s41592-021-01282-5. PMC9255697 34725479

[261] van Staalduinen J, van Staveren T, Grosveld F, Wendt KS. Live-cell imaging of chromatin contacts opens a new window into chromatin dynamics. Epigenetics Chromatin. 2023;16(1):27. 10.1186/s13072-023-00503-9. PMC10288748 37349773

[262] Xiao Z, Sun Y. Illuminating the genome: emerging approaches in CRISPR-Cas live-cell imaging. Nucleic Acids Res. 2026;54(2):gkaf1540. 10.1093/nar/gkaf1540. PMC12805899 41538311

[263] Zhu Y, Moerner WE, Qi LS. CRISPR-Cas-based live cell imaging of genome dynamics. Nat Rev Genet. 2026;27(8):622–41. 10.1038/s41576-026-00949-z. 41951915

[264] Brandão HB, Gabriele M, Hansen AS. Tracking and interpreting long-range chromatin interactions with super-resolution live-cell imaging. Curr Opin Cell Biol. 2021;70:18–26. 10.1016/j.ceb.2020.11.002. PMC8364313 33310227

[265] Zhou H, Han Y, Bai Y, Zuo Y, He W, Guo F. Diff-SE: A Diffusion-Augmented Contrastive Learning Framework for Super-Enhancer Prediction. J Chem Inf Model. 2025;65(14):7789–99. 10.1021/acs.jcim.5c01005. 40613852

[266] MacPhillamy C, Alinejad-Rokny H, Pitchford WS, Low WY. Cross-species enhancer prediction using machine learning. Genomics. 2022;114(5):110454. 10.1016/j.ygeno.2022.110454. 36030022

[267] Hu W, Li Y, Wu Y, Guan L, Li M. A deep learning model for DNA enhancer prediction based on nucleotide position aware feature encoding. iScience. 2024;27(6):110030. 10.1016/j.isci.2024.110030. PMC11167433 38868182

[268] He W, Zhou H, Zuo Y, Bai Y, Guo F. MuSE: A deep learning model based on multi-feature fusion for super-enhancer prediction. Comput Biol Chem. 2024;113:108282. 10.1016/j.compbiolchem.2024.108282. 39561516

[269] Sun Y, Liu S, Chen L, Zhou Z, Yin X, Shi Y, et al. AI-driven discovery of dual antiaging and anti-AD therapeutics via PROTAC target deconvolution of a super-enhancer-regulated axis. Sci Adv. 2025;11(47):eadz9283. 10.1126/sciadv.adz9283. PMC12629202 41259504

[270] Wang A, Xia H, Li J, Diao P, Cheng J. Development of a novel prognostic signature derived from super-enhancer-associated gene by machine learning in head and neck squamous cell carcinoma. Oral Oncol. 2024;159:107016. 10.1016/j.oraloncology.2024.107016. 39244857

[271] Long F, Zhang H, Zhang S, Dong Z, Huang X, Hu R. Machine learning applications in the detection and treatment of esophageal cancer. Discov Oncol. 2026;17(1):952. 10.1007/s12672-026-05477-0. PMC13319654 42334709

[272] Bono A, La Monica G, Alamia F, Tocco D, Lauria A, Martorana A. B.R.E.A.S.T. Breast canceR enhanced AI-supported therapy: a new interpretable proteomics-driven machine learning framework for therapy response prediction in breast cancer. Int J Mol Sci. 2026;27(12):5163. 10.3390/ijms27125163. PMC13299364 42352887

[273] Wang X, Xiong D, Cui S, Duan B, Hung Y, He J, et al. Artificial intelligence for precision oncology from phenotyping and drug discovery to clinical translation. Discov Oncol. 2026;17(1):504. 10.1007/s12672-026-04864-x. PMC13036062 41857437

[274] Soroudi S, Jaafari MR, Arabi L. Lipid nanoparticle (LNP) mediated mRNA delivery in cardiovascular diseases: Advances in genome editing and CAR T cell therapy. J Control Release. 2024;372:113–40. 10.1016/j.jconrel.2024.06.023. 38876358

[275] Masarwy R, Stotsky-Oterin L, Elisha A, Hazan-Halevy I, Peer D. Delivery of nucleic acid based genome editing platforms via lipid nanoparticles: Clinical applications. Adv Drug Deliv Rev. 2024;211:115359. 10.1016/j.addr.2024.115359. 38857763

[276] Lyu P, Lu B. New Advances in Using Virus-like Particles and Related Technologies for Eukaryotic Genome Editing Delivery. Int J Mol Sci. 2022;23(15):8750. 10.3390/ijms23158750. PMC9369418 35955895

[277] Hołubowicz R, Gao F, Du SW, Rodrigues Menezes C, Zhang J, Hołubowicz MW, et al. Scalable purification enables high-quality virus-like particles for therapeutic translation. J Biol Chem. 2025;301(12):110946. 10.1016/j.jbc.2025.110946. PMC12753231 41241097

[278] He X, Zeng S, Wen Y, Yang T, Huang C, Li Y, et al. Covalent Recruitment of NEDD4 for Targeted Protein Degradation: Rational Design of Small Molecular Degraders. J Am Chem Soc. 2025;147(25):21512–25. 10.1021/jacs.4c18083. 40506832

[279] Taha EA, Lee J, Hotta A. Delivery of CRISPR-Cas tools for in vivo genome editing therapy: Trends and challenges. J Control Release. 2022;342:345–61. 10.1016/j.jconrel.2022.01.013. 35026352

[280] Lei J, Yang K, Cao W, Qi S, Du X, Li H, et al. Pancreatic-targeted lipid nanoparticles based on organ capsule filtration. Nature. 2026;652(8108):220–9. 10.1038/s41586-026-10158-7. 41741655

[281] Zhang X, Yang Y, Zhang M, Fan K, Huang Z, Zhang Y, et al. Multi-omics identification of MSI2 as a super-enhancer-driven vulnerability in MYCN-amplified neuroblastoma. J Transl Med. 2026;24(1):886. 10.1186/s12967-026-08296-7. PMC13359825 42143291

[282] Geyer FH, Ritter A, Kinn-Gurzo S, Faehling T, Li J, Jarosch A, et al. Comprehensive DSRCT multi-omics analyses unveil CACNA2D2 as a diagnostic hallmark and super-enhancer-driven EWSR1::WT1 signature gene. Cancer Commun. 2025;45(6):702–8. 10.1002/cac2.70015. PMC12187571 40088092

[283] Yu Y, Zhang X, Ma X, Ren J, Zhang J, Zhu L, et al. Identification of super-enhancer-based biomarkers for predicting survival and immunotherapy efficacy in colorectal cancer. J Cancer. 2026;17(2):338–58. 10.7150/jca.119265. PMC12825423 41584044

[284] Gao T, Zheng Z, Pan Y, Zhu C, Wei F, Yuan J, et al. ScEnhancer: a single-cell enhancer resource with annotation across hundreds of tissue/cell types in three species. Nucleic Acids Res. 2022;50(D1):D371–9. 10.1093/nar/gkab1032. PMC8728125 34761274

[285] Yang F, Liang G, Shi H, Yan L. The clinical diagnostic value of the super-enhancer-associated long noncoding RNA RP11-803D5.4 and AC005592.2 in colorectal cancer. Curr Med Chem. 2026;33(3):659–75. 10.2174/0109298673346788250108080648. 39871572

